# Immunological effects of reduced mucosal integrity in the early life of BALB/c mice

**DOI:** 10.1371/journal.pone.0176662

**Published:** 2017-05-01

**Authors:** Katja Maria Bendtsen, Camilla Hartmann Friis Hansen, Łukasz Krych, Kerstin Skovgaard, Witold Kot, Finn Kvist Vogensen, Axel Kornerup Hansen

**Affiliations:** 1 Section of Experimental Animal Models, Department of Veterinary and Animal Sciences, Faculty of Health and Medical Sciences, University of Copenhagen, Copenhagen, Denmark; 2 Department of Food Science, Faculty of Sciences, University of Copenhagen, Copenhagen, Denmark; 3 Section for Immunology and Vaccinology, National Veterinary Institute, Technical University of Denmark, Copenhagen, Denmark; 4 Department of Environmental Science, Aarhus University, Roskilde, Denmark; Kurume University School of Medicine, JAPAN

## Abstract

Certain stimuli at the gut barrier may be necessary in early life to establish a proper balance of immune tolerance. We evaluated a compromised barrier in juvenile mice in relation to microbiota and local and systemic immunity. BALB/c mice were treated with a low dose of dextran sulfate sodium (DSS) with or without ampicillin and lipopolysaccharide (LPS) to clarify the importance of microbial antigens and interaction between microbial-associated patterns and toll-like receptors. The barrier breach resulted in increased plasma LPS, which was highest in mice treated simultaneously with ampicillin. Adding LPS in the food reduced its levels in plasma. Regulatory T cells were acutely increased in mesenteric lymph nodes (MLN) and spleen during DSS treatment regardless of simultaneous ampicillin treatment. In contrast, NK T and NK cells decreased in MLN and in spleen. This acute DSS effect was reflected in fold changes of *haptoglobin* and *Il1a* in colon, and this was also more pronounced in mice simultaneously treated with ampicillin. On day 1 post-treatment, major upregulations of *Ifng*, *Foxp3*, *Il1b*, *Il2*, and *Il6* genes in colon were only observed in the mice simultaneously treated with ampicillin. A two-fold upregulation of colonic *Foxp3* and *Il1a* was evident 25 days post-treatment. DSS skewed the microbiota in favor of Gram negative phyla. Therefore, increased permeability induced tolerogenic immunity independent of microbiota, and this was enhanced by LPS stimulation.

## Introduction

As first proposed by Strachan in 1989 and since debated as the hygiene hypothesis [1], a sufficient antigenic stimulus is likely necessary in early life to obtain protective regulatory immunity against later life inflammatory diseases. This hypothesis has been further developed into the “old friends” hypothesis [2] claiming that increasing incidences of inflammatory diseases are more precisely due to an increasing lack of diversity in the gut microbiota over generations. A relevant example of a widely discussed risk factor is caesarean section and the enhanced risk of immune-mediated diseases. By this cleaner-than-intended delivery mode, the newborn may not readily be exposed to the maternal microbiota during vaginal birth resulting in delayed or insufficient colonization [3] and reduced regulatory immunity [4].

The presence of mucosa-associated bacteria has been related to a healthy gut environment. Bacteria express microbial-associated molecular patterns (MAMPs) which depending on age, antigen type and -load, amount of immunosuppressive cytokines, as well as the presence of antimicrobial peptides and host immune status, interact with host pattern-recognizing receptors (PRRs), such as the important Toll-like receptors (TLRs) [5]. *Akkermansia muciniphila* are mucus-decomposing Gram-negative bacteria of the *Verrucomicrobia* phylum. These bacteria show reverse proportionality with inflammatory bowel disease (IBD) [6] and appendicitis [7] in humans, they are reduced in abundance in children with autism [8], and they have been linked to increased tolerance and decreased type 1 diabetes incidence in non-obese diabetic mice [9]. Colonization of germ-free mice have shown that *A*. *muciniphila* prefer mucus-rich locations such as the cecum, and are closely affiliated with the ileum and colon epithelium. In comparison to *Lactobacillus plantarum* that upregulate host lipid metabolism, *A*. *muciniphila* upregulates genes involved in antigen presentation, and, therefore, may play a significant role as a commensal tolerance establisher [10,11]. Inoculation of gram-positive *Clostridia* species in conventional mice in early life, mediates TGF_β_ production, increases T_reg_ cells, and provide protection from colitis later in life through TLR stimulation to [12]. Colonization with the eight symbiotic strains of the altered Schaedler flora [13] expands the T_reg_ cell population in the colonic lamina propria, but not in the regional lymph nodes or systemically [14].

Dextran sulfate sodium (DSS) is a sulfated polysaccharide, which in solutions of 2–5% in the drinking water, is used for induction of colitis in rodents. It has been suggested that DSS with its effects on mucus layer integrity on gene level [15] and on *ex vivo* penetrability [16] induces mucosal permeability and microbial translocation that in fact precede inflammation [17]. DSS exposure induces severe colonic injury with systemic morbidity in mice deficient of MyD88, an essential downstream adaptor molecule for most MAMPs, but this was completely prevented by antibiotics followed by oral administration of the Gram-negative TLR4 ligand lipopolysaccharide (LPS), and the Gram-positive TLR2 ligand lipoteichoic acid [18]. In this study, IL-6 and TNF-α were upregulated in DSS-treated wild-type mice, while they were downregulated in DSS-treated MyD88 deficient mice [18]. The compromised mucosal integrity caused by a low, non-inflammatory dose of DSS may, therefore, if given shortly in early life, allow an increased mucosal immune cell-MAMP interaction, and thereby mimic the mucin degrading effects of *A*. *muciniphila*.

We evaluated the effect of a low-dose DSS-induced compromised intestinal barrier on immune cells, intestinal mRNA transcripts and gut microbiota (GM) in juvenile mice. We hypothesized that this DSS treatment would compromise the mucosal barrier sufficiently for the gut bacterial antigens to induce a regulatory tolerant immune response even without clinical inflammation. Five weeks old BALB/c mice were treated for seven days with 1.5% DSS, alone (full microbiota) or in combination with ampicillin (suppressed microbiota) to investigate the necessity of bacterial stimuli during the barrier breach, and in combination with LPS to specify the need for TLR4 signaling.

## Materials and methods

The study was conducted in agreement with Directive 2010/63/EU of the European Parliament and of the Council of 22 September 2010 on the protection of mice used for scientific purposes, and the Danish Animal Experimentation Act (LBK 474 15/05/2014). The study was approved by The Animal Experiments Inspectorate under The Ministry of Environment and Food of Denmark (License: 2007-561-1434 C3).

### Mice and experimental design

120 female BALB/cAnNBomTac mice, three weeks old at arrival, were randomly allocated to 20 standard cages (type 1290, Techniplast, Italy) with six mice in each cage. The 20 cages were randomized into four groups with five cages in each. Mice were purchased with a health report according to Taconic’s routine health monitoring program. In the unit, sentinel mice were serologically tested for mouse hepatitis virus, reovirus type 3, Theiler's virus (GDVII), Sendai virus, minute virus of mice, mouse parvovirus, rotavirus (EDIM) and Clostridium piliforme (Biodoc Diagnostics, Hannover Germany). Fecal samples from cages were tested in-house for endoparasites (Fecalyzer, Kruuse A/S, Marbjerg, Denmark). Full microbiota 16S sequencing was done in-house on samples from a high number of mice from the studies, as described below. None of the assays revealed any agents to be reported according to FELASA guidelines for health monitoring.

From arrival to treatment in week five, all mice were fed a standard Altromin 1324 diet (Altromin, Germany). During week five, one group was treated with DSS in the drinking water (1.5%, dextran sulfate sodium, cat. no. 160110, lot no. M2709, MW = 36000–50000, MP Biomedicals, USA). Two groups received ampicillin in the drinking water from arrival (1 g/L Ampivet Vet, 250 mg/ml, Boehringer Ingelheim, Germany) and were then during week five either simultaneously treated with DSS, or with DSS and a diet containing added lipopolysaccharide (Rodent diet D12070602, 40.8 mg/kg LPS, Brogaarden, Denmark). The other treatment groups received a corresponding control diet (Rodent diet D12450J). After one week of treatment all groups received standard diet and tap water (S2 Fig). Housing conditions were: a 7 a.m.–7 p.m. light/dark cycle, 440 lx light intensity, relative humidity 55% +/−10%, and a 20–24°C temperature. The mice were provided with Aspen bedding (Tapvei, Estonia) with supplement of Enviro-dri and Alpha-Nest nesting material (SSP, USA), Shepherd’s Shacks (regular, SSP, USA), and Aspen chew blocks (Tapvei, Estonia).

The mice were killed on days 3 and 5 during treatment and on days 1, 7, and 25 post-treatment. From these time points samples of blood, feces, liver, ileum and colon were taken. On day 3 during treatment and on day 25 post-treatment, the spleen and mesenteric lymph nodes were sampled. For histology, samples of ileum and colon were preserved on day 3 during treatment and on day 1 and 25 post-treatment. Samples from day 3 of treatment and from days 1 and 25 post-treatment were analyzed.

The mice were sedated with a 1:1 Hypnorm/Midazolam mixture (0.315 mg/ml fentanyl and 10 mg/ml fluanisone (Hypnorm, VetPharma, Denmark) and 5 mg/ml midazolam (Roche, Denmark)), blood was obtained from the orbital sinus, and the mice were killed by cervical dislocation.

### Gene expression

Ileum and colon samples were placed in RNAlater upon removal (Qiagen) and kept at -20°C until processing. The samples were transferred to gentleMACS^™^ M tubes (MACS, Miltenyi Biotec, Lund, Sweden) containing QIAzol (Qiagen, Ballerup, Denmark) and homogenized using the gentleMACS^™^ Dissociator (MACS, Miltenyi Biotec). Total RNA was extracted with chloroform (Merck, VWR, Herlev, Denmark) and ethanol (Kementyl, VWR), and subsequently isolated using the RNeasy Lipid kit (Qiagen) including on-column digestion of genomic DNA using the RNase-Free DNase set (Qiagen) according to manufacturer’s protocol. Total RNA concentration and purity were measured using a NanoDrop ND-1000 spectrophotometer (Saveen and Werner AB, Sweden), and the RNA integrity was assessed using the Agilent Bioanalyzer 2100 and RNA 6000 Nano Kit (Agilent Technologies).

An RNA Integrity Number (RIN) from 1–10 was assigned to each total RNA sample by the Agilent Bioanalyzer, with 10 being non-degraded RNA. RINs for ileum samples were between 7.8–10.0 (av. 9.3) and 6.6–9.8 (av. 8.9) for colon samples (S3 Table). First-strand cDNA synthesis was performed with 500 ng total RNA per sample (water for non-template-controls/non-RT-controls) using the QuantiTect Reverse Transcription Kit (Qiagen) according to manufacturer’s protocol. For assay validation, two cDNA synthesis reactions were prepared per sample. Prior to preamplification, the cDNA was diluted 1:6 in low EDTA TE-buffer (VWR—Bie & Berntsen, Herlev). Preamplification was performed using TaqMan PreAmp Master Mix (Applied Biosystems). Stocks of 200 nM primer mix were prepared combining equal concentrations of all primers used in the present study. TaqMan PreAmp Master Mix (5 μl) was mixed with 2.5 μl 200 nM stock primer mix and 2.5 μl diluted cDNA, and incubated at 95°C for 10 minutes followed by 16 cycles of 95°C for 15 sec. and 60°C for 4 min. Before qPCR, preamplified cDNA was diluted at least 1:4 in low EDTA TE-buffer (VWR—Bie & Berntsen). Primers were designed using Primer3 (http://frodo.wi.mit.edu/), synthesized at Sigma-Aldrich (Denmark), as previously described [19] (primers, gene names and abbreviations are presented in S4 Table). Amplification efficiencies, dynamic range, and specificity were estimated based on four separate dilution series of pooled cDNA and melting curve profiles. Quantitative PCR was performed in 48.48 Dynamic Array Integrated Fluidic Circuits (Fluidigm, CA, USA.), combining 48 samples with 48 primer sets for 2304 simultaneous qPCR reactions as previously described [20]. Non-template controls (NTC) were included to monitor any potential problems with non-specific amplification or sample contaminations. Non-reverse transcriptase controls were included to assess potential genomic DNA contamination.

Expression data (Cq values) were acquired using the Fluidigm Real-Time PCR Analysis software 3.0.2 (Fluidigm) and exported to GenEx (MultiD, Göteborg) for data pre-processing including correction for PCR efficiency for each primer assay individually, normalizing to highly stable reference genes, and using technical repeats of cDNA. For the ileum samples, ribosomal protein L13a (Rpl13a), beta-2 microglobulin (B2m) and beta-actin (Actb) were identified as the most stable expressed reference genes out of 8 candidates using GeNorm [21] and NormFinder [22]. This also applied to the colon samples, with addition of the reference gene beta-glucuronidase (Gusb). For each primer assay, the mean relative expression level was set to 1 in the group with the lowest level of expression, and all other groups were adjusted accordingly during log-transformation (log2) to linear scale.

### Cell isolation and flow cytometry

The number of T_reg_ and NK/NK T cells in the spleen and mesenteric lymph nodes (MLN) were counted for assessment of general and local immune cell recruitment. The tissues were harvested and immediately placed in cold PBS on ice. Cells were isolated from spleen and MLN collected immediately upon euthanasia by aseptically squeezing the fresh organs in PBS between two microscope slides and subsequently passing the suspension through a 70 μm cell strainer. Flow cytometric analyzes of NK, NK T, and FoxP3+ T_reg_ cells were done as previously described [9]. All antibodies were purchased from eBiosciences. The analyses were performed using an Accuri C6 flow cytometer (Accuri Cytometers Inc, Ann Arbor, MI).

### LPS

LPS content was measured with a fluorescence excitation/emission ELISA system (Pyrogene Recombinant Factor C Endotoxin Detection Assay, 50-658U, Lonza, Germany) in plasma diluted 1:1000 with MgCl_2_ (10mMol, S50-641, Lonza, Switzerland), established by the product inhibition test according to manufacturer’s protocol. The diluted samples were heat-inactivated in a 70°C water bath for 10 minutes before proceeding, as recommended by the manufacturer. The fluorescence is proportional to the endotoxin concentration in a double log scale and is linear in the 0.005 to 5.0 EU/ml range.

### Histology

Ileum and colon samples were preserved in either formalin or Carnoy’s fixative for 24 hours and kept in ethanol until they were embedded in paraffin, sliced and HE and Alcian-Blue/PAS dyed.

### Body weight, food and water consumption

Minimum once a week the weights of mice, food, and water bottles were noted and accumulated body weights along with food and water consumption were calculated.

### Bacterial DNA

Fecal pellets were collected upon handling into an autoclaved microtube, placed on wet ice and stored at -80°C. Bacterial DNA was extracted with QIAamp DNA Stool Mini Kit 51504 (Qiagen). Quality and quantity of DNA were spectrophotometrically evaluated (NanoDrop 1000, Thermo Scientific, USA) and stored at -80°C. Samples were cleaned (PowerClean^®^ Pro DNA Clean-Up Kit, Mobio Laboratories, USA), and spermine was added to prevent DSS inhibiting the polymerase (unpublished manuscript).

### High throughput sequencing

The fecal microbiota of 45 mice was determined using tag-encoded 16S rRNA gene MiSeq-based (Illumina, CA, USA) high throughput sequencing. Cellular DNA extraction, DNA storage condition, and sequencing library preparation steps were conducted as previously described [23]. The raw dataset containing pair-ended reads with corresponding quality scores was merged and trimmed using settings as previously mentioned. Quantitative Insight Into Microbial Ecology (QIIME) open source software package [24] (1.7.0 and 1.8.0) was used for subsequent analysis steps. Purging the dataset from chimeric reads and constructing *de novo* Operational Taxonomic Units (OTU) was conducted using the UPARSE pipeline [25]. The green genes (13.8) 16S rRNA gene collection was used as a reference database [26].

Principal coordinate analysis (PCoA) plots were generated with the Jackknifed Beta Diversity workflow based on 10 distance metrics calculated using 10 subsampled OTU tables. The number of sequences taken for each jackknifed subset was set to 85% of the sequence number within the most indigent sample. Analysis of similarities (ANOSIM) was used to evaluate group differences using weighted and unweighted UniFrac [27] distance metrics that were generated based on rarefied (10,000 reads/sample) OTU tables. The relative distribution of the genera registered was calculated based on subsampled OTU tables (summarized to the genus level).

Alpha diversity measures expressed with an observed species (sequence similarity 97% OTUs) value were computed for rarefied OTU tables (10,000 reads/sample) using the alpha rarefaction workflow. Differences in alpha diversity were determined using a t-test-based approach employing the non-parametric (Monte Carlo) method (999 permutations) implemented in the compare alpha diversity workflow.

ANOVA was used to determine qualitative quantitative (relative abundance) association of OTUs with the given group. These were calculated based on 1000 subsampled OTU tables rarefied to an equal number of reads (10,000 per sample) and summarized to the genus level. Both the *p*-value and the conservative FDR-corrected *p*-value for multiple comparisons are reported.

### General statistics

qPCR: Fold change was calculated based on relative Cq values, and the criteria of <-2 or >2 were applied. Differentially expressed mRNA transcripts were tested with multiple t-tests with Benjamini-Hochberg correction. Flow cytometry and LPS: Normalized data was tested with ANOVA or unpaired two-tailed t-test. Correlations: Permutation based method was used to initially calculate r and p between relative counts of operational taxonomic units and immunological data, and Pearson correlation in Graphpad Prism 7.02 was used for graphical presentation.

## Results

### LPS leak to the plasma was dependent on antigen presence

**Fig 1:**
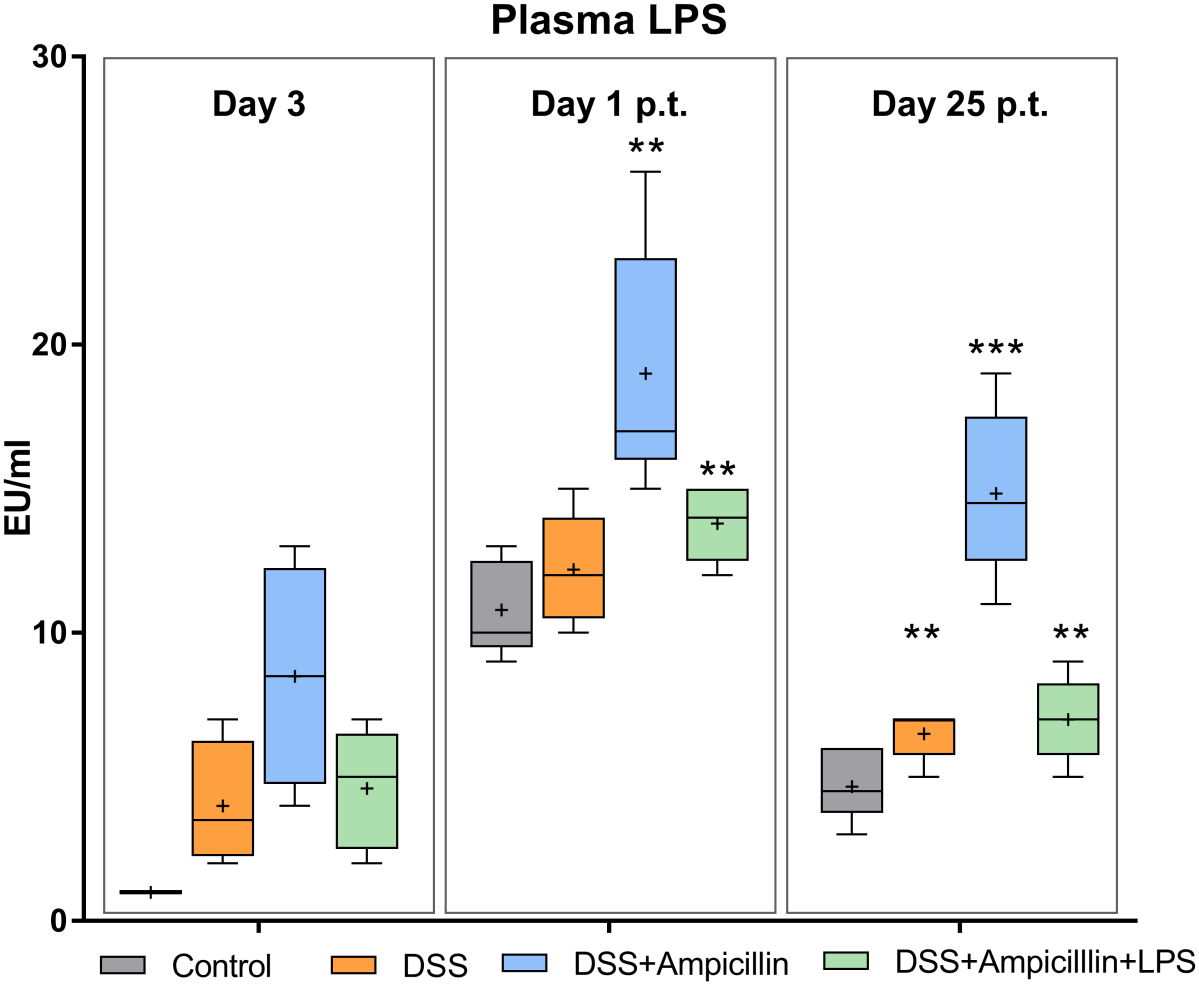
Plasma lipopolysaccharides. Lipopolysaccharides measured by ELISA in plasma of mice during, as well as 1 and 25 days post-treatment with 1.5% dextran sulfate sodium (DSS), 1g/L ampicillin and/or diet containing 40.8 mg/kg lipopolysaccharides (LPS). Box and whisker plots, + shows mean; whiskers: min.-max., n = 5–6 per group (p.t. = post-treatment, p-values: ** = <0.01; *** = p<0.001, compared to control group. All controls from day 3 were only 50% above detection limit of the assay).

DSS treatment increased the amount of LPS in plasma during treatment, demonstrating a breach in the intestinal barrier. This was also evident in a bacterially suppressed state, which as hypothesized shows a direct effect of DSS on the gut. Addition of LPS in the diet during DSS treatment did not increase plasma LPS further (Fig 1). In fact, there seemed to be a protective effect of TLR4 signaling on the gut barrier, as plasma LPS was improved in mice receiving dietary LPS compared to the mice only treated with ampicillin. The compromised barrier resulted in increased plasma LPS in all treatment groups compared to the control group. However, in all mice, except for those receiving both DSS and ampicillin, the levels were decreased when the treatment was terminated (Fig 1).

### Acute increase in T_reg_ cells and late decrease in NK and NK T cells

**Fig 2:**
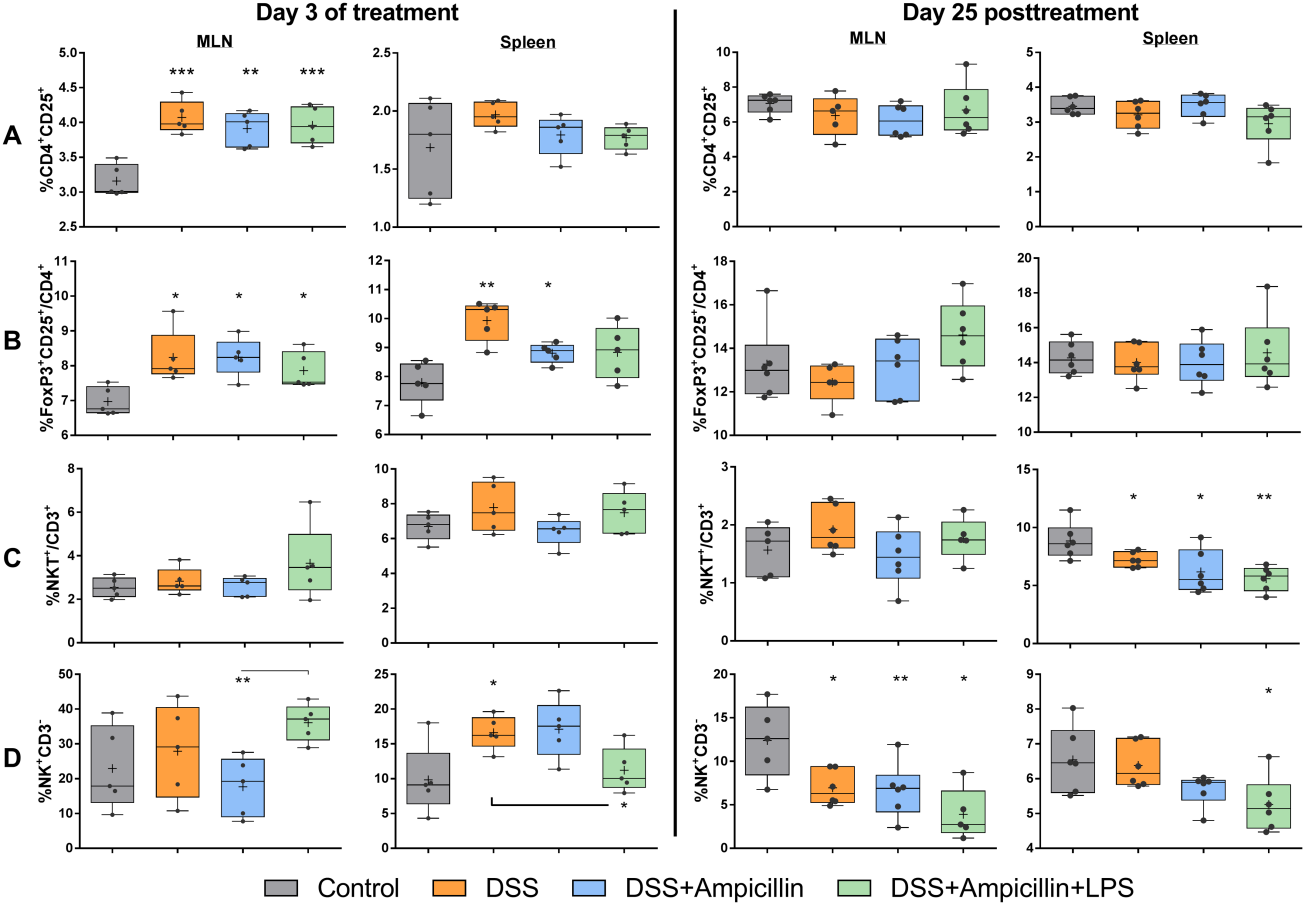
Immune cell flow cytometry. Flow cytometric analysis of cells isolated from mesenteric lymph nodes (MLN) and spleen in mice during and 25 days post-treatment with 1.5% dextran sulfate sodium (DSS), 1g/L ampicillin and/or diet containing 40.8 mg/kg lipopolysaccharides (LPS). Percentages of CD4^+^CD25^+^ activated T helper cells present among all lymphocytes (A), FoxP3^+^ T_reg_ cells among CD4^+^ T cells (B), NKT^+^ among all T cells (C) and NK cells among all lymphocytes (D) are shown. Box and whisker plots, + shows mean; whiskers: min.-max., n = 5–6 per group as shown by dots (p-values: * = p<0.05; ** = <0.01; *** = p<0.001, no line: compared to control group).

On day 3 of treatment, MLN of all treatment groups contained more activated T helper cells compared to the control group. This was not evident in the spleen (Fig 2A).

The fractions of CD4^+^FoxP3^+^CD25^+^ T_reg_ cells were significantly increased in both MLN and spleen in DSS-treated groups on day 3; even if treated simultaneously with ampicillin which indicates that microbes were not responsible for this effect (Fig 2B). 25 days post-treatment this effect had vanished. In contrast, there was a late effect on NK T cells and NK cells which were significantly decreased on day 25 post-treatment in all treatment groups, most significantly for mice treated with DSS, ampicillin and LPS in combination (Fig 2C+2D).

### Microbiota suppression increased the compromising effect on the barrier

The mRNA transcripts of 35 genes (S4 Table) were evaluated and considered relevant when at least 2-fold up or downregulated in treated mice compared to control mice (Table 1 and S1 Table). The largest fold regulations were observed on day 1 post-treatment, when *Hp* and *Il1a* were upregulated in the colon of DSS mice with a larger fold change in mice simultaneously treated with ampicillin. A 7-9-fold upregulation of *Ifng* and 2-8-fold upregulations of *Foxp3*, *Il1b*, *Il2*, and *Il6* in colon were only observed in mice simultaneously treated with ampicillin, suggesting an increased barrier-compromising effect of microbiota suppression. Interestingly, *Muc1* in colon was upregulated in DSS mice simultaneously treated with ampicillin, but not when supplemented with LPS. On day 3 of treatment a 2-fold upregulation of *Il2* together with a 2-3-fold downregulation in *Il10* and *Muc2* were observed in the colon of DSS mice simultaneously treated with ampicillin. On day 25 after treatment there was a small late effect in DSS mice simultaneously treated with ampicillin with a 2-fold upregulation of *Foxp3* and *Il1a* in colon.

**Table 1:** Fold change <-2 or >2 of mRNA transcripts.

COLON*	Day 3 of treatment**			**Day 1 pt**			**Day 25 pt**		
	DSS^a^	DSS+Amp^b^	DSS+Amp+LPS^c^	DSS	DSS+Amp	DSS+Amp+LPS	DSS	DSS+Amp	DSS+Amp+LPS
*Cxcl9*					4.10	**7.17**			
*Defa*		2.09					3.99		
*Foxp3*					**2.89**	2.45		**2.14**	
*Hp*	12.13	5.34	4.62	**3.92**	5.83	**4.30**			
*Ifng (1)*					7.88	**8.32**			
*Ifng (2)*					9.03	**9.44**			
*Il23*	3.25								
*Il10*		**-3.73**	**-2.65**						
*Il1a*				**2.02**	**4.57**	**3.31**		**2.09**	
*Il1b*					**2.89**	**3.55**			
*Il2*		**2.35**	2.19		**5.03**	**2.75**			
*Il6*		7.97	3.61		15.91	**8.32**			
*Muc1 (1)*				2.86	**3.20**	2.11		2.93	2.00
*Muc1 (2)*				2.53	**2.07**				
*Muc2 (1)*		**-4.63**	**-2.07**		-2.09				
*Muc2 (2)*		**-2.59**							
**ILEUM**	**Day 3 of treatment**			**Day 1 pt**			**Day 25 pt**		
	DSS	DSS+Amp	DSS+Amp+LPS	DSS	DSS+Amp	DSS+Amp+LPS	DSS	DSS+Amp	DSS+Amp+LPS
*Cxcl10*					**-3.10**	**-2.37**			
*Cxcl9*					**-3.93**	**-3.35**			
*Ifng (1)*	-3.54	-3.97	-5.84		**-12.11**	**-8.58**			-4.17
*Ifng (2)*					**-6.25**	**-6.52**			-3.90
*Il10*	-2.17	-2.41	**-3.31**		**-4.24**	**-2.96**			
*Il18*								**2.15**	2.02
*Il6*						**2.72**			
*Retnlb*	-4.26	**-39.78**	**-11.03**	**-12.17**				-2.58	

mRNA from mouse ileum and colon during, as well as 1 and 25 days post treatment with 1.5% dextran sulfate sodium (DSS), 1g/L ampicillin and/or diet containing 40.8 mg/kg lipopolysaccharides (LPS). Control = 1. Bold: statistically significant (t-test with FDR). Light to dark blue: decreasing fold change <-2; Pink to red: increasing fold change >2.

*See S4 for gene names.

**See S2 Fig for study design.

^a^DSS; dextran sulfate sodium,

^b^Amp; ampicillin,

^c^LPS; lipopolysaccharide.

### DSS treatment decreased gut microbiota diversity with a skewing towards Gram-negative phyla

There were clear time point differences and development in the bacterial composition within each treatment group (S1 Fig). Ampicillin-treated mice on days 3 and 1 post-treatment were excluded due to the low DNA concentration in the isolates, confirming suppression of bacteria. Due to the absence of qualitative or quantitative differences in bacterial composition, these groups were combined as one on day 25 post-treatment. On days 3 and 1 post-treatment the species diversity was significantly reduced in DSS-treated mice compared to control mice (p<0.01). This discrepancy was lost on day 25 post-treatment, when only the ampicillin-treated groups had significantly reduced diversity compared to the control group. Mice only treated with DSS regained their diversity (alpha diversity measures expressed with observed species index).

**Fig 3:**
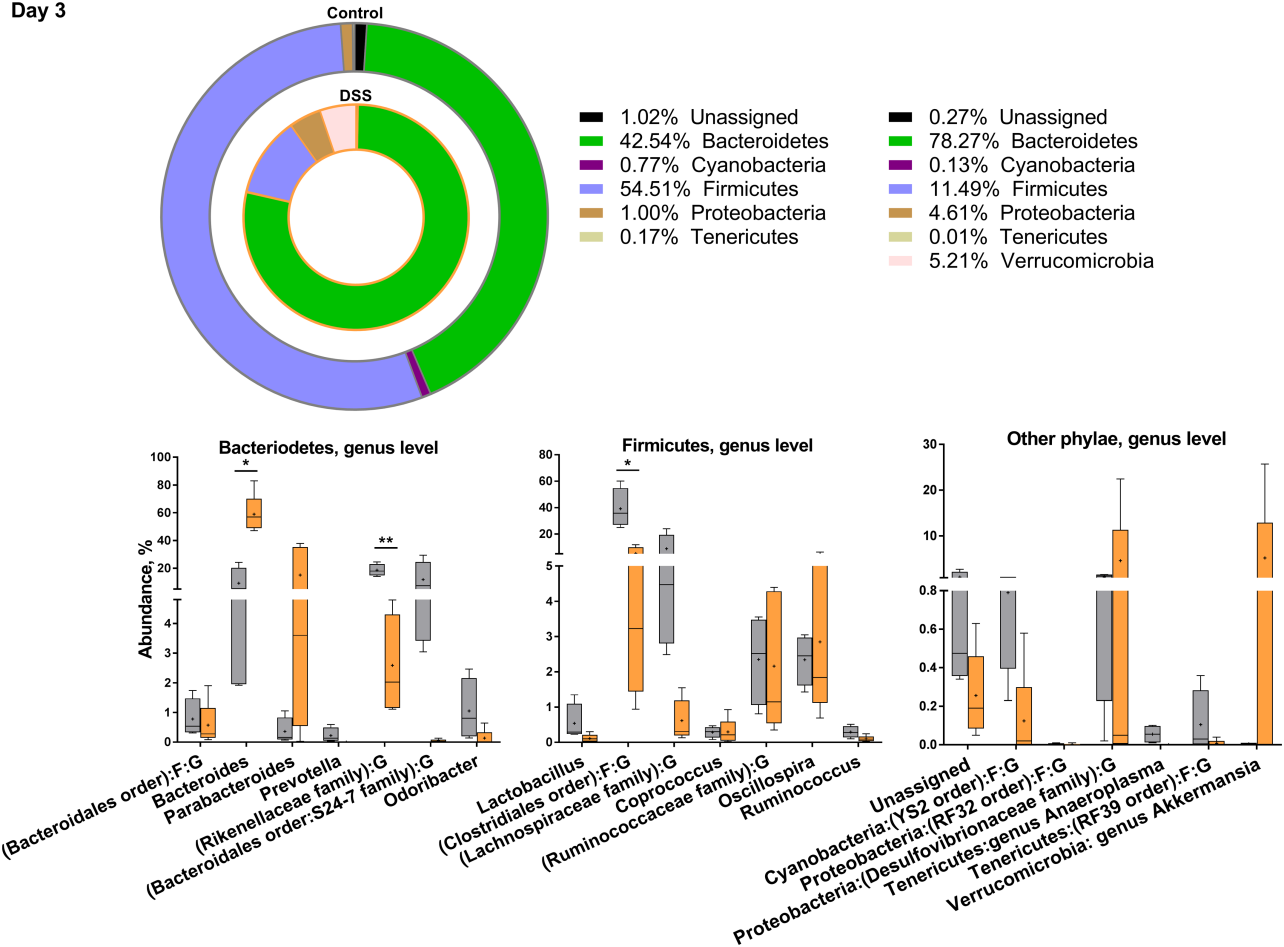
Distribution of the 20 most abundant phyla on day 3 of treatment. Overview of relative phylogenetic distribution and details of variation down to genus level of gut microbiota of untreated control mice and mice during treatment with 1.5% dextran sulfate sodium (DSS). There were too few reads from DSS+Ampicillin and DSS+Ampicillin+LPS treated mice on day 3 and day 1 post-treatment. Verrucomicrobia amount on day 3 and day 1 post-treatment is for one mouse only. See S2 Table for analysis of variance.

**Fig 4:**
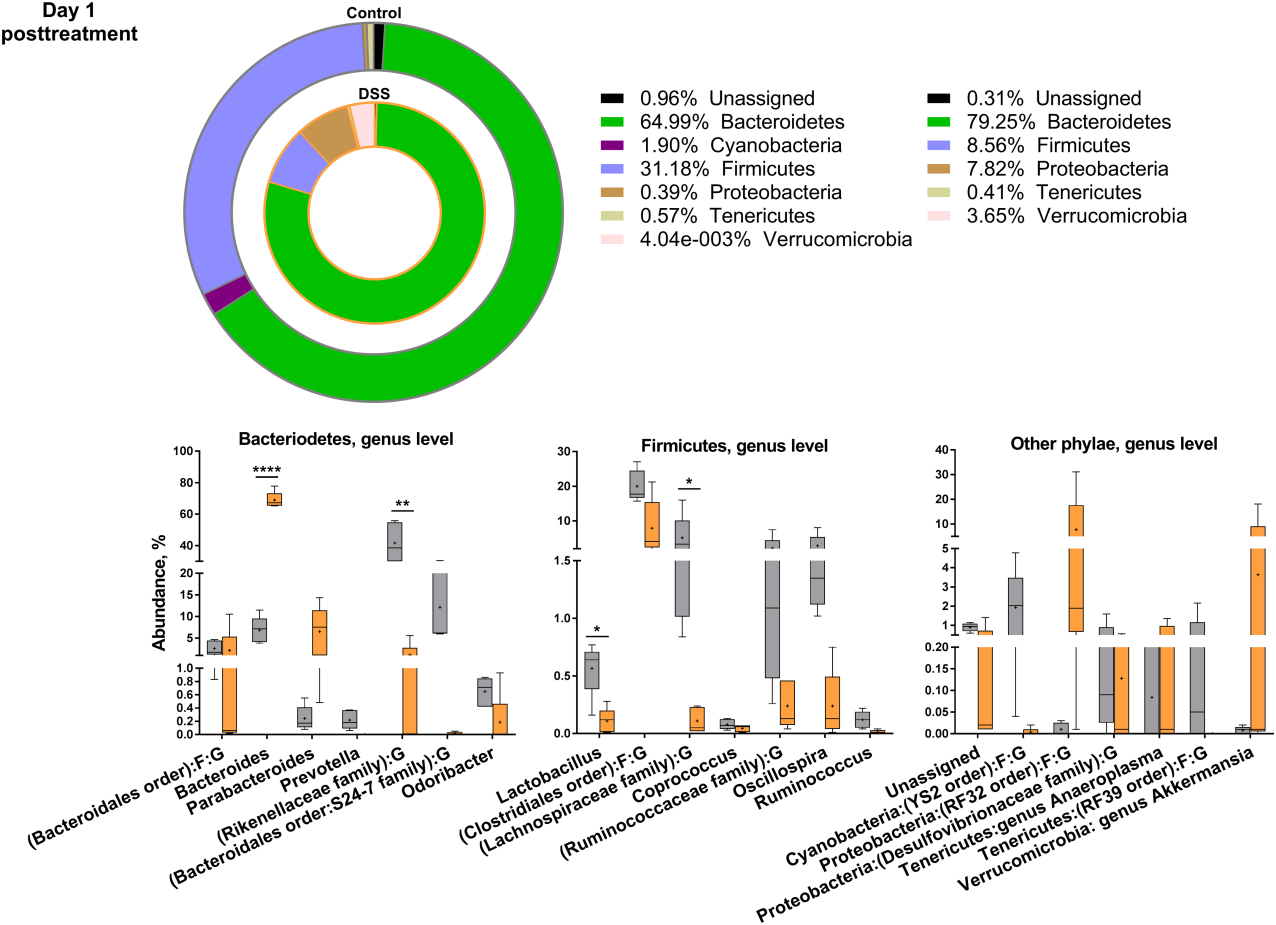
Distribution of the 20 most abundant phyla on day 1 post-treatment. Overview of relative phylogenetic distribution and details of variation down to genus level of gut microbiota of untreated control mice and mice during treatment with 1.5% dextran sulfate sodium (DSS). There were too few reads from DSS+Ampicillin and DSS+Ampicillin+LPS mice on day 3 and day 1 post-treatment. Verrucomicrobia amount on day 3 and day 1 post-treatment is for one mouse only. See S2 Table for analysis of variance.

**Fig 5:**
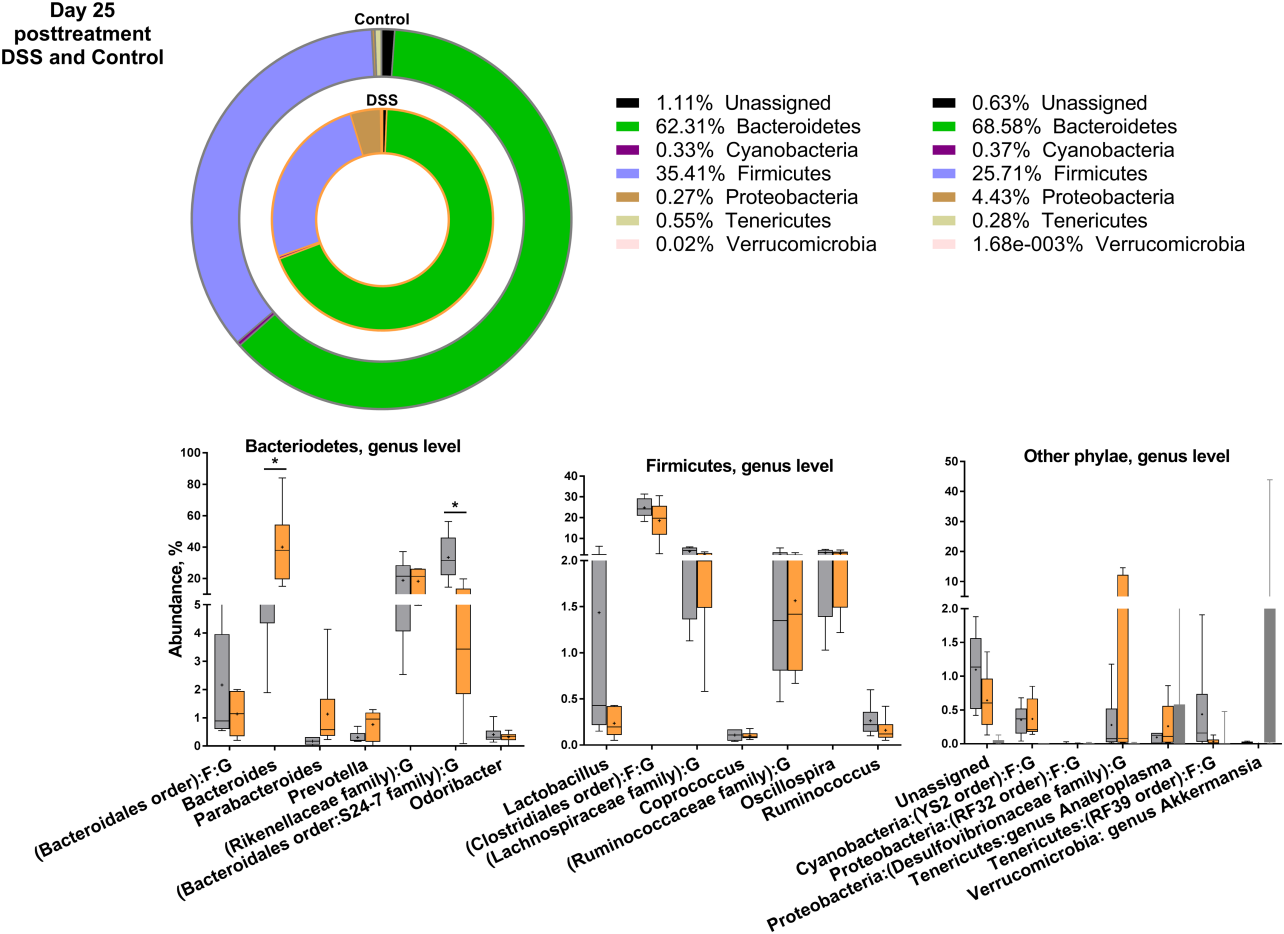
Distribution of the 20 most abundant phyla on day 25 post-treatment (Control and DSS). Overview of relative phylogenetic distribution and details of variation down to genus level of gut microbiota of untreated control mice compared to mice 25 days post-treatment with 1.5% dextran sulfate sodium (DSS). See S2 Table for analysis of variance.

**Fig 6:**
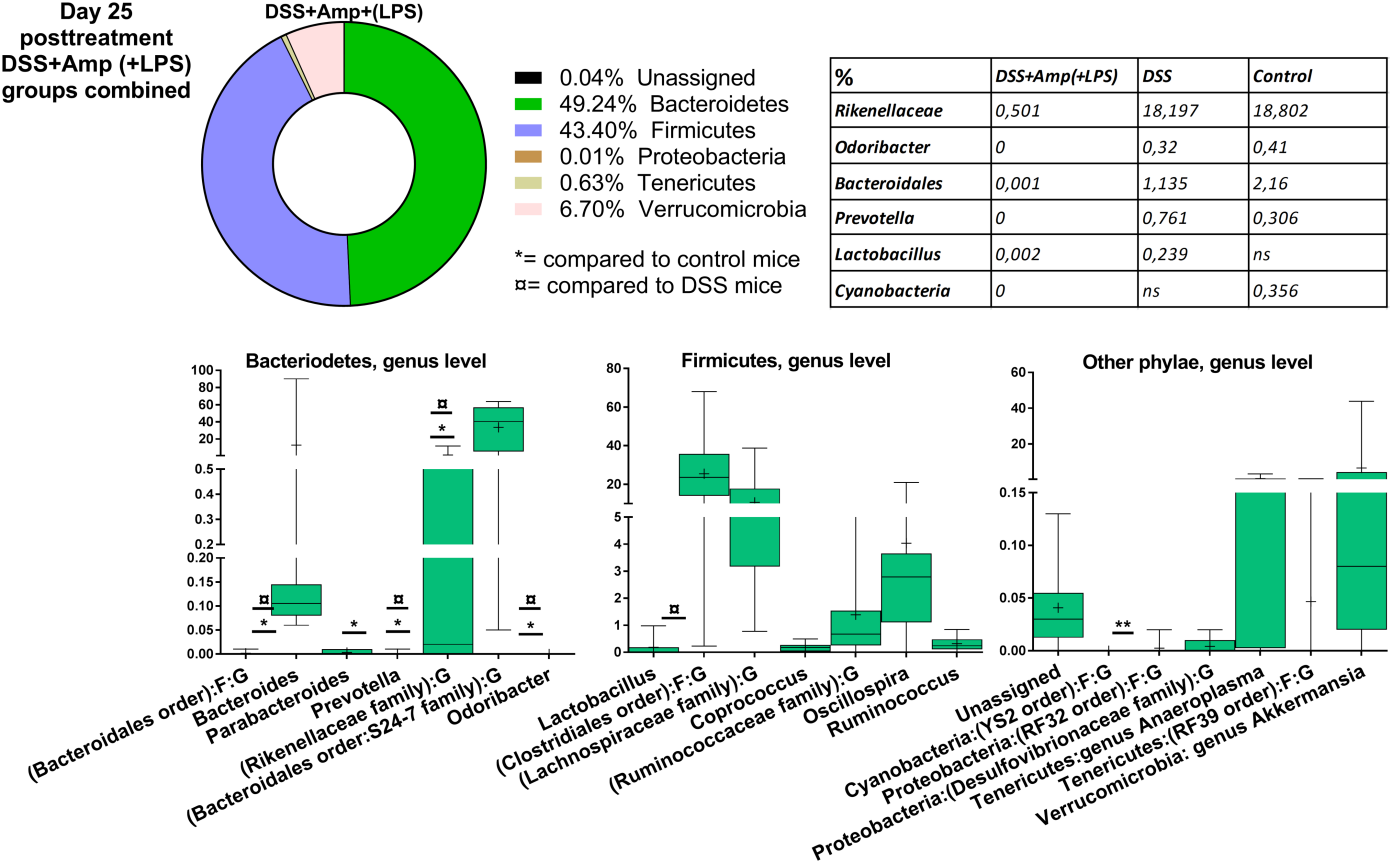
Distribution of the 20 most abundant phyla on day 25 post-treatment (DSS+Ampicillin (+LPS) groups combined). Overview of relative phylogenetic distribution and details of variation down to genus level of gut microbiota of untreated control mice compared to mice 25 days post-treatment with 1.5% dextran sulfate sodium (DSS), 1g/L ampicillin (Amp) and/or diet containing 40.8 mg/kg lipopolysaccharides (LPS). There were too few reads from DSS+Amp and DSS+Amp+LPS mice on day 3 and day 1 post-treatment. As no qualitative or quantitative differences in bacterial composition were found between DSS+Amp mice and DSS+Amp+LPS on day 25 post-treatment, these groups were combined for this time point. See Fig 5 for control group and DSS group. See S2 Table for analysis of variance.

Overall, in an overview of the 20 most abundant species at each time point, the DSS treatment tended to skew the bacterial composition in favor of Gram-negative phyla (Figs 3–6 and S2 Table). DSS treatment significantly decreased the family of Rikenellaceae (control = 18%, DSS = 2%), and increased the Gram-negative *Bacteroides* genus (control = 9%, DSS = 58%) on day 3 of treatment, both belonging to the Bacteriodetes phylum. In turn, DSS treatment decreased the Gram-positive Firmicutes genus *Candidatus Arthromitus* also known as segmented filamentous bacteria (SFB) (control = 0.2%, DSS = 0.004%), and the Firmicutes order of Clostridiales (control = 39%, DSS = 5%) (Fig 3). On day 25 post-treatment, *Bacteroides* spp. were still significantly increased in the DSS-treated mice (Fig 5), and a member of the Bacteroidales order, S24-7, decreased as described for day 3 of treatment and day 1 post-treatment (Fig 4). For most other significantly different bacteria, the DSS mice on day 25 post-treatment (Fig 5) resembled the control animals. In contrast, Rikenellaceae and Firmicutes families were together with several Bacteriodetes families still reduced in the ampicillin-treated mice, compared to control and DSS mice (Fig 6).

### OTUs correlated with immunological changes

**Fig 7:**
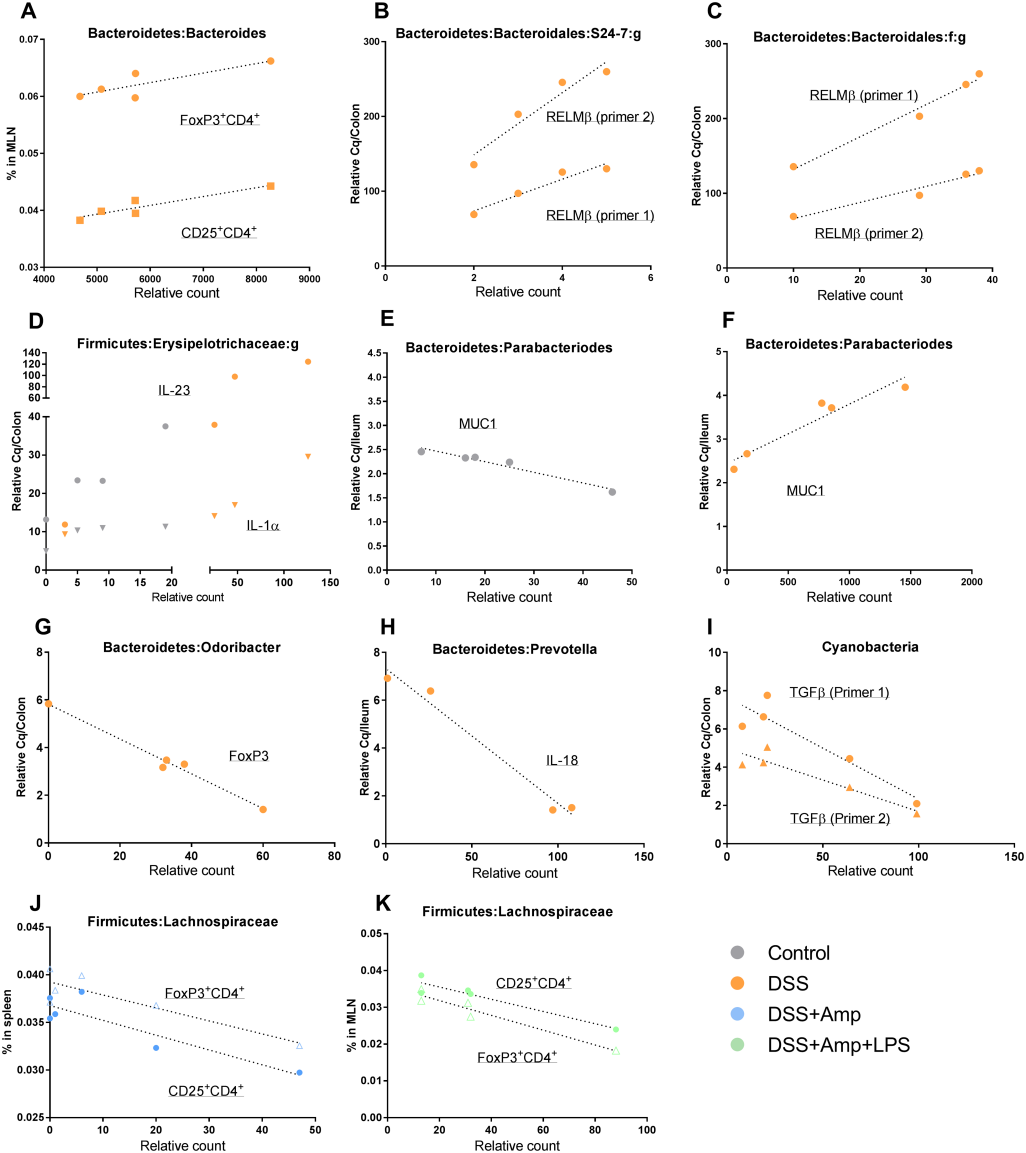
Correlations of relative counts of some operational taxonomic units and parameters. OTUs from gut microbiota and immunological parameters of untreated control mice and mice treated with 1.5% dextran sulfate sodium (DSS) alone or in combination with ampicillin (Amp) and lipopolysaccharides (LPS). The permutation based method was used to calculate r and p. Depicted p-values are FDR corrected. A-D: Day 3 of treatment. A. FoxP3^+^CD4^+^; p = 0.0485, r = 0.88, CD25^+^CD4^+^; p = 0.0087, r = 0.93. B. RELMβ (primer 1); p = 0.0022, r = 0.97, RELMβ (primer 2); p = 0.00009, r = 0.97. C. RELMβ (primer 1); p = 0.0131, r = 0.91, RELMβ (primer 2); p = 0.0100, r = 0.92. D. IL-23; p = 0.0391, r = 0.92, IL1α; p = 0.0012, r = 0.97. E+F: Day 1 post-treatment. E: p = 0.0046, r = 0.97. F: p = 0.0105, r = 0.96. G-K: Day 25 post-treatment. G. p = 0.0010, r = -0.9914. H. p = 0.0124, r = -0.9876. I. TGFβ (primer 1); p = 0.0244, r = -0.9251, TGFβ (primer 1); p = 0.0209, r = -0.9325. J. FoxP3^+^CD4^+^; p = 0.0208, r = -0.8799, CD25^+^CD4^+^; p = 0.0162, r = -0.8942. K. FoxP3^+^CD4^+^; p = 0.0154, r = -0.9449, CD25^+^CD4^+^; p = 0.0079, r = -0.9648.

On day 3 of DSS-treatment *Bacteriodetes* spp. increased and this correlated with increase of T_reg_ in mesenteric lymph nodes (MLN) (Fig 7A), and with upregulation in RELMβ transcripts (Fig 7B+7C). Interestingly, a lower amount of the Firmicutes family Erysipelotrichaceae correlated with lower amounts of IL-23 and IL-1α transcripts in control mice, compared to increased amounts of Erysipelotrichaceae and IL-23 and IL-1α in DSS-treated mice (Fig 7D). Similarly, on day 1 following DSS treatment, MUC1 expression and relative counts of *Parabacteroides* spp. belonging to the *Bacteriodetes* genus correlated reversely in DSS treated mice compared to control (Fig 7E+7F). *Odoribacter* and *Prevotella* spp. correlated negatively to FoxP3 and IL18 on day 25 after DSS treatment, and Cyanobacteria was negatively correlated to TGFβ (Fig 7G–7I). Interestingly, for the two groups treated with DSS and ampicillin (with or without LPS), there was a negative correlation between T_reg_ cells and relative counts of the Lachnospiraceae family 25 days post-treatment (Fig 7J+7K).

### No clinical effect of low-dose DSS in the absence of microbiota suppression

Clinically, the DSS-only treated mice were statistically indistinguishable from the control mice, with only small perturbations in food and water intake mildly resembling the pattern of the ampicillin-treated groups immediately following treatment cessation (Figure D in S1 File). The ampicillin-treated mice showed small transient weight loss, decreased food intake and a significant increase in water intake compared to control mice (Figures B+C+D in S1 File). In turn, the mice were monitored on a daily basis for clinical signs of colitis, and the ampicillin-treated mice were examined on day 1 post-treatment, as a few mice were observed with bloody stools from day five of treatment. When the treatment ended, all mice from these groups returned to normal, showing no signs of colitis. Water intake differed slightly between the groups (Figures B+D in S1 File). This led to slight differences in DSS intake (Figure A in S1 File) ranging from 14.1 to 21.3 mg DSS per gram body weight in the individual mice, but it has previously been shown that DSS load only acts in a straight dose-dependent manner in a critical load above 30 mg per gram body weight [28]. Histological sections showed no apparent aberrations on any day of sampling.

## Discussion

In this study, young mice were treated with a low dose of DSS for seven days. Mice treated with DSS alone did not exhibit any clinical signs and were similar to control mice in weight and food and water intake.

Some mice were simultaneously treated with ampicillin, and others were, in addition to DSS, treated with LPS in the diet. The ampicillin-treated mice with no LPS added were found to continuously leak more LPS to the plasma, which may suggest that TLR4 signaling actually protects the gut barrier and impedes LPS leakage from the gut. It could, therefore, be of interest to study the impact of this early life low-dose DSS treatment with and without dietary LPS in TLR4 deficient mice. In turn, *Muc1* in colon was only significantly upregulated in DSS mice simultaneously treated with ampicillin on day 1 post-treatment, whereas DSS treatment alone or supplementation with LPS did not produce a *Muc1* upregulation. The upregulation could be interpreted as a compensatory mechanism in response to DSS removal of mucus, as previously shown [29], and hence, LPS or full GM protected from removal of mucus, thereby supporting a healthy barrier. This is in line with the study in which LPS and lipoteichoic acid alone protected against inflammation [18]. In our study, the amount of mRNA coding for the LPS receptor gene *Tlr4* (and of the *Tlr1* gene) was significantly regulated, but below our fold criterion, hence the biological relevance of this result was questioned and not included.

T_reg_ cells were increased on day 3 of treatment in MLN and spleen of all DSS-treated groups, and as T_reg_ cells and *Foxp3* mRNA transcripts were also increased in ampicillin-treated mice, these effects seemed to be independent of GM, and hence, point at a direct local and systemic effect of this barrier compromise. Our study further showed that DSS treatment promotes Gram-negative phyla, especially Bacteriodetes, which correlated with T_reg_ cells in the MLN and mRNA transcripts of the barrier-related Retnlb (Relmβ) on day 3 of DSS treatment. Interestingly, *Parabacteroides* spp. were inversely correlated to colonic MUC1 expression in control and DSS treated mice on day 1 following DSS treatment, with a positive correlation in DSS treated mice. In turn, 25 days after DSS treatment *Odoribacter* spp. and *Prevotella* spp. were negatively associated with FoxP3 and IL-18 expression. The *Bacteriodetes* family has previously been linked to DSS treatment [30,31], and thus it is not unrealistic that DSS increases the chance of LPS/TLR-receptor interaction leading to tolerogenic cell responses.

In our study, an acute effect of DSS was reflected in fold changes of *Hp* and *Il1a* in colon on day 3 of treatment, and this effect was more pronounced in mice also treated with ampicillin. On day 1 post-treatment, major upregulation of *Ifng*, *Foxp3*, *Il1b*, *Il2*, and *Il6* in colon was only observed in bacterially depleted mice in which LPS leakage was also highest. Interestingly, a 2-fold upregulation of *Foxp3* and *Il1a* in colon was evident later on, which was seemingly a local effect not apparent at the more systemic cellular level. Others have also reported a classical Th1 response in BALB/c mice with increasing upregulation in *Il12*, *Ifng*, *Il1*, and *Tnfα* as dosage of DSS increased (2.5, 5 and 7.5%) [32], as well as slight increases of IL-6, IL-17, and IL-4 [33] were observed.

The bacterial presence in the gut after antibiotic treatment must logically depend on treatment regimen and recolonization time, in combination with handling and housing status. In this study, the mice were treated with ampicillin from arrival at three weeks until six weeks of age (with simultaneous treatment with DSS and LPS during week five), and then evaluated 25 days post-treatment (S2 Fig). Samples were collected on day three in week five, and day one in week six, and it was not possible to yield enough reads due to the very low DNA concentration of the isolates, at least confirming successful depletion at these time points. The results of others using the same dose of ampicillin range from elimination of all aerobic bacteria [34], increase in Streptococcaceae and Lactobacillaceae [35], and a general decrease of all bacteria [18], to extended effects in combination with other antibiotics (see [36]). Consequently, the GM was most likely only heavily suppressed, despite the very low DNA concentration in the fecal samples of the ampicillin-treated mice. One potential follow-up study would, therefore, be to treat germ-free mice with DSS and LPS, which in relation to the present study would have the advantage that the mice would be fully devoid of bacteria. The advantage of the present approach using ampicillin is that all mice have experienced similar neonatal priming with bacteria at birth, and the microbiota-suppressed group is only suppressed during the experimental period. A study with germ-free mice adds up both effects of neonatal priming and current microbial stimulation [37]. Furthermore, the control group would either have to be ex-germ-free inoculated mice, which may not necessarily establish a full GM [38], or conventional mice subjected to bacterial priming at birth. DSS lowered the microbial diversity during and immediately following treatment, but this was regained later on, whereas decreased diversity was still present in ampicillin-treated mice. The early effect of DSS on T_reg_ cells and the late effect on NK/NK T cells were also present in ampicillin-treated mice suggesting that it is the effect on the barrier rather than the effect on the microbes that is of importance. It is very likely that increased contact with dietary antigens during DSS treatment promoted the oral tolerance independently of the microbial antigens present.

Five weeks of age may be too late to permanently affect immune parameters, and studies focusing on the pre-weaning period or pre-birth conditions have successfully been able to modulate immune parameters showing a long-term effect, for instance by maternal diet [39] and GM colonization of GF mice [38], on T_reg_ and NK T cell populations. However, studies of probiotic intervention at later ages also reported successful outcomes [40,41], and the immune system of rodents is not fully developed until sexual maturity [42]. The question remains if and when an early life gut barrier breach is positively associated with health. Studies of obesity and type 2 diabetes point to a negative systemic effect of what is called a “leaky gut” where a high level of LPS in the blood leads to constant low-grade inflammation [43]. Naturally, it is necessary to distinguish between biological leakiness early and late in life, and the adult homeostatic condition in between. Perhaps immune system priming is especially important during exposed stages of life, such as during the early life immune system immaturity, when education is essential for further development, or during the old life immune system exhaustion with constant need for “nudging”.

Our data show similarity to DSS colitis induction in the acute stage with a low inflammatory response and a transient T_reg_ increase. The decreased levels of NK and NK T cells later in life may indicate relocation to tissues or a sign of regulatory change. In fact, it has been established that an important interplay exists between T_reg_ and NK T cells in regulatory immunity, as NK T cells are able to modulate T_reg_ function by IL-2 secretion, which was also found upregulated in the present study, and inversely, NK T cell proliferation, cytokine expression and cytotoxic activity can be inhibited by T_reg_ cells [44]. The innate NK cells are also highly interrelated with T_reg_ cells, in disease states and during normal homeostasis. During pregnancy, these two cell types seem to work together to create tolerance, and in cancer a high number of T_reg_ cells, presumably suppressing NK cells, often correlates with poor disease outcome [45]. Hence, the increase in T_reg_ cells during early DSS treatment is possibly a factor in the later observed decrease in NK and NK T cells. NK T cells are regulated by the intestinal environment [46], and they have been reported increased in the colon of GF mice from weaning and through life compared to specific pathogen-free mice, leading to increased severity of oxazolone-induced colitis, which was reversed if neonatal GF mice were colonized with a conventional GM [47].

We hypothesized that a short low-dose DSS treatment in five weeks old BALB/c mice would compromise the mucosal barrier sufficiently to induce a regulatory tolerant immune response with transient inflammation ranging from none to mild. We were able to show a regulatory cellular response acutely, while the effects later in life were mainly characterized by decreases in NK and NK T cells. An inflammatory challenge later in life, such as the oxazolone-induced colitis model [48], or the use of a disease model with an early onset such as type 1 diabetes, would be useful to further evaluate the importance of both permanent and immediate effects of such early life manipulations.

## Supporting information

S1 Fig Projections of the qualitative and quantitative gut microbiota composition. Principal coordinate analysis (PCoA) plots of gut microbiota composition of mice during, and 1 and 25 days post treatment with 1.5% dextran sulfate sodium (DSS), 1g/L ampicillin and/or diet containing 40.8 mg/kg lipopolysaccharides (LPS). Plots are based on unweighted (A, C, E) and weighted (B, D, F) distance matrices calculated from 10 rarefied (10,000 reads) OTU tables showing clear separation between groups. DSS+Amp and DSS+Amp+LPS treated mice on day 3 and 1 post-treatment were excluded due to the low DNA concentration in the isolates, confirming depletion of bacteria. As no qualitative or quantitative differences in bacterial composition were found between DSS+Amp mice and DSS+Amp+LPS on day 25 post-treatment, these groups were combined for this time point. P-values of similarity (ANOSIM) between treatment groups: Control vs DSS day 3; r = 0.987, p = 0.010 (A) and r = 0.850, p = 0.011 (B). Control vs DSS day 1 pt.; r = 0.989, p = 0.003 (C) and r = 1.000, p = 0.006 (D). Day 25 pt: Control vs DSS; r = 0.518, p = 0.003 (E) and r = 0.324, p = 0.007 (F); Control vs Amp. groups; r = 0.658, p = 0.001 (E) and ns (F).
(TIF)

S2 Fig Study design. From arrival to treatment in week five, all mice were fed a standard diet. During week five, one group was treated with 1.5% dextran sulfate sodium (DSS) in the drinking water. Two groups received ampicillin (1g/L) in the drinking water from arrival and were then co-treated during week five with either DSS, or with DSS and diet containing added lipopolysaccharide (LPS) (40.8 mg/kg LPS). The other treatment groups received corresponding control diet. After one week of treatment all groups received standard diet and tap water. Mice were killed on day 3 and 5 during treatment and on day 1, 7, and 25 post-treatment. From these time points samples of blood, feces, liver, ileum and colon were taken. On day 3 during treatment and on day 25 pt., the spleen and mesenteric lymph nodes were sampled (= FACS). For histology, samples of ileum and colon were preserved on day 3 during treatment, and on day 1 and 25 pt. Samples from day 3 of treatment and from day 1 and 25 pt. were analyzed (= S). P-values of similarity (ANOSIM) within groups over time (day 3-day 1 pt/day 1 pt-day 25 pt): Control unweighted = ns, weighted = p<*, DSS unweighted = p<*/**, weighted = ns/p<* (exact values not shown).
(TIF)

S1 File DSS load, body weight, food and water intake. Data of mice during, and 1 and 25 days post treatment with 1.5% dextran sulfate sodium (DSS), 1g/L ampicillin and/or diet containing 40.8 mg/kg lipopolysaccharides (LPS). Figure A. Average DSS load (mg/g BW, calculated from cage level water intake per mouse in cage) in the different treatment groups during treatment, in week 5 (day 32–39). Ampicillin in the water increased the water intake, and thereby DSS load in groups co-treated with ampicillin. Figures B+C. Food and water intake divided in periods. Animals were treated from day 32–39. Day 21–28: First week of acclimatization (ampicillin-treated groups were acclimatized to ampicillin water from arrival). Day 36–49: From day 5 of treatment to 10 days post-treatment. Day 36–63: From day 5 of treatment to day 25 post-treatment. Significantly increased water intake in groups receiving ampicillin (B), and food intake was significantly increased in the first week of acclimatization (C). Figure D. Overview curves of overall cumulative weight gain, water and food intake. Food and water intake was measured on cage level and divided with the number of animals per cage. The mice were weighed individually, but for these graphs the average weight of the animals in one cage was used to calculate the average intake per mouse. Control and DSS show similar patterns characterized by decrease in food intake, rise in water intake after treatment period (shaded area). DSS+Amp and DSS+Amp+LPS had lower water and food intake during treatment compared to control and DSS animals, with changes continuing in the period following treatment cessation, where the mice consumed less food and lost weight, and elicited increased water intake.
(PDF)

S1 Table P-values for mRNA transcripts with <2 or >2 fold change. (DOCX)

S2 Table Analysis of variance of gut microbiota. (DOCX)

S3 Table RNA Integrity. (DOCX)

S4 Table Primers. (DOCX)
