# Supplementary figures and images for: Immunological effects of reduced mucosal integrity in the early life of BALB/c mice
Source: PLoS One. 2017 May 1;12(5):e0176662. doi: 10.1371/journal.pone.0176662 (PMC5411035; doi:10.1371/journal.pone.0176662)

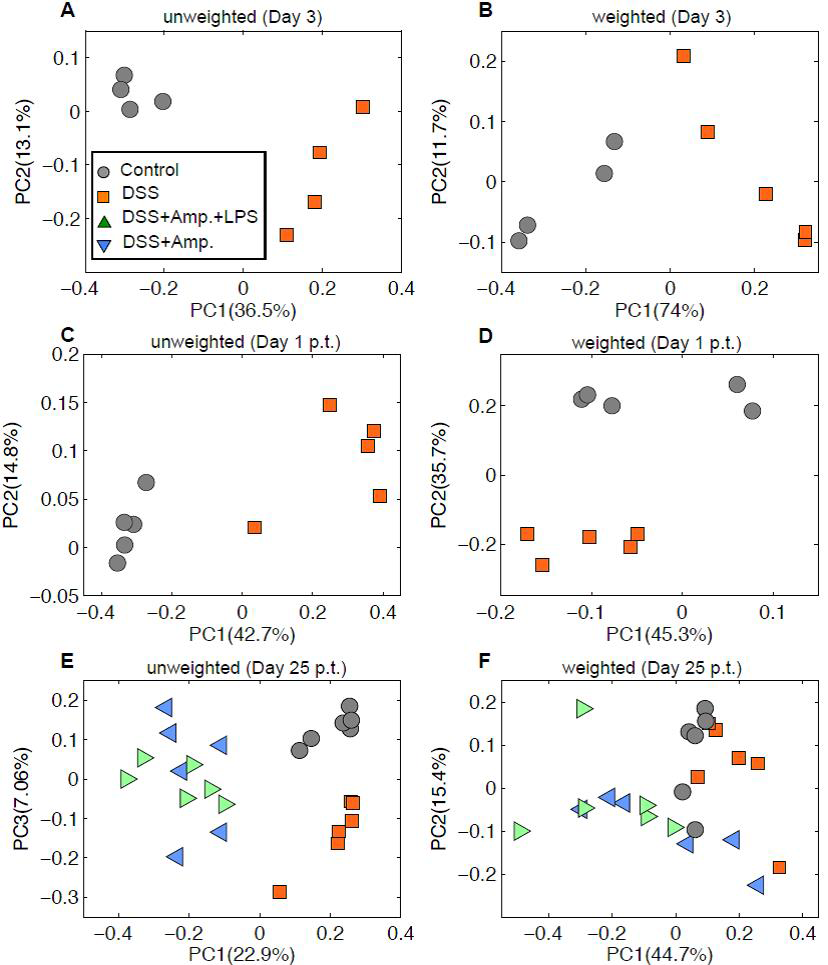

Supplement: S1 Fig — Principal coordinate analysis (PCoA) plots of gut microbiota composition of mice during, and 1 and 25 days post treatment with 1.5% dextran sulfate sodium (DSS), 1g/L ampicillin and/or diet containing 40.8 mg/kg lipopolysaccharides (LPS). Plots are based on unweighted (A, C, E) and weighted (B, D, F) distance matrices calculated from 10 rarefied (10,000 reads) OTU tables showing clear separation between groups. DSS+Amp and DSS+Amp+LPS treated mice on day 3 and 1 post-treatment were excluded due to the low DNA concentration in the isolates, confirming depletion of bacteria. As no qualitative or quantitative differences in bacterial composition were found between DSS+Amp mice and DSS+Amp+LPS on day 25 post-treatment, these groups were combined for this time point. P-values of similarity (ANOSIM) between treatment groups: Control vs DSS day 3; r = 0.987, p = 0.010 (A) and r = 0.850, p = 0.011 (B). Control vs DSS day 1 pt.; r = 0.989, p = 0.003 (C) and r = 1.000, p = 0.006 (D). Day 25 pt: Control vs DSS; r = 0.518, p = 0.003 (E) and r = 0.324, p = 0.007 (F); Control vs Amp. groups; r = 0.658, p = 0.001 (E) and ns (F). (TIF) [file pone.0176662.s001.tif]

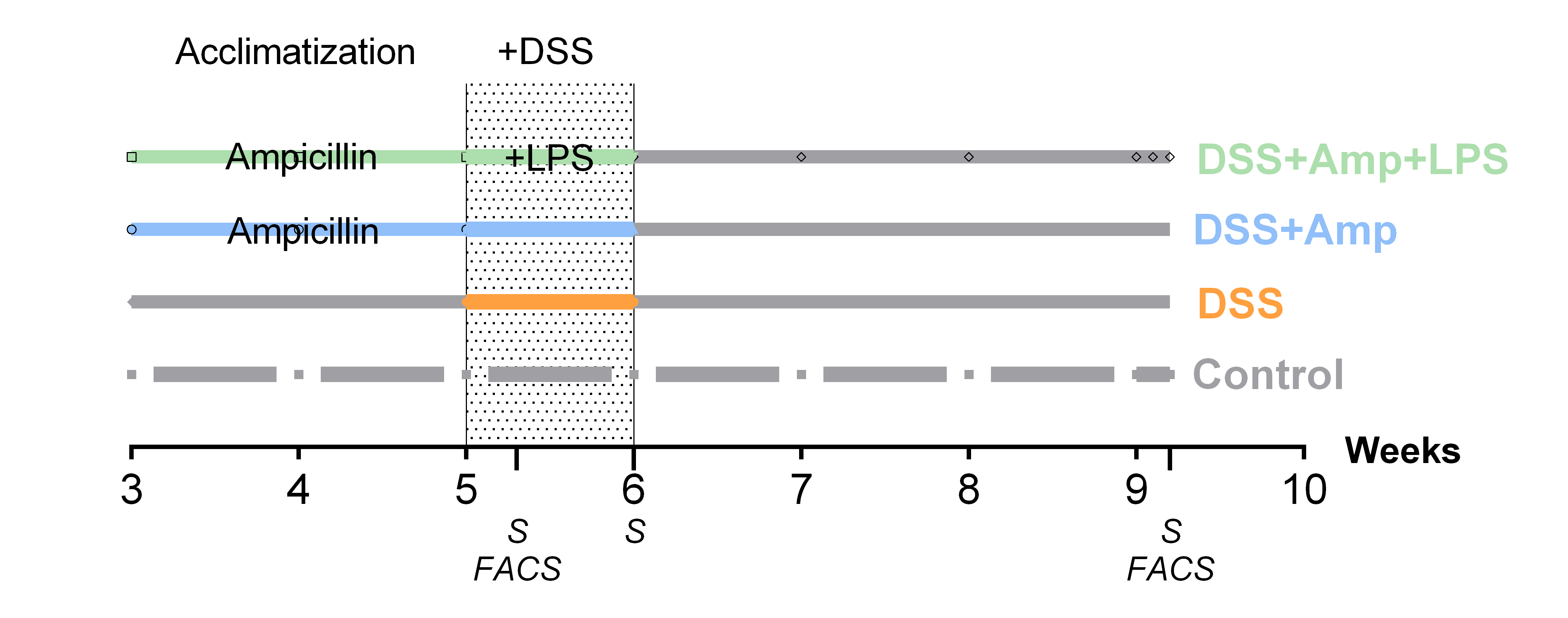

Supplement: S2 Fig — From arrival to treatment in week five, all mice were fed a standard diet. During week five, one group was treated with 1.5% dextran sulfate sodium (DSS) in the drinking water. Two groups received ampicillin (1g/L) in the drinking water from arrival and were then co-treated during week five with either DSS, or with DSS and diet containing added lipopolysaccharide (LPS) (40.8 mg/kg LPS). The other treatment groups received corresponding control diet. After one week of treatment all groups received standard diet and tap water. Mice were killed on day 3 and 5 during treatment and on day 1, 7, and 25 post-treatment. From these time points samples of blood, feces, liver, ileum and colon were taken. On day 3 during treatment and on day 25 pt., the spleen and mesenteric lymph nodes were sampled (= FACS). For histology, samples of ileum and colon were preserved on day 3 during treatment, and on day 1 and 25 pt. Samples from day 3 of treatment and from day 1 and 25 pt. were analyzed (= S). P-values of similarity (ANOSIM) within groups over time (day 3-day 1 pt/day 1 pt-day 25 pt): Control unweighted = ns, weighted = p<*, DSS unweighted = p<*/**, weighted = ns/p<* (exact values not shown). (TIF) [file pone.0176662.s002.tif]

A

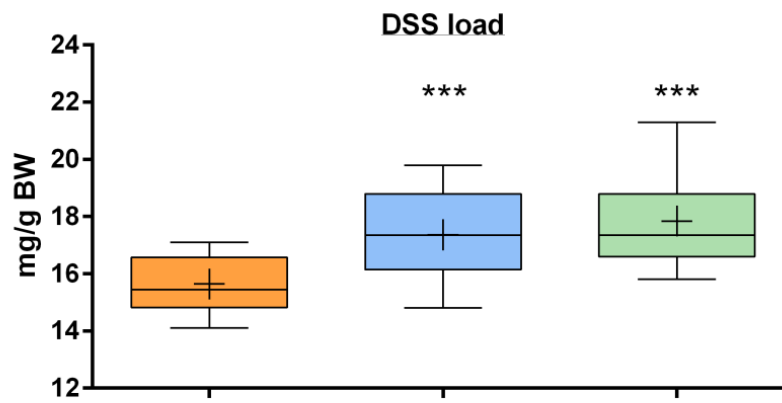

B

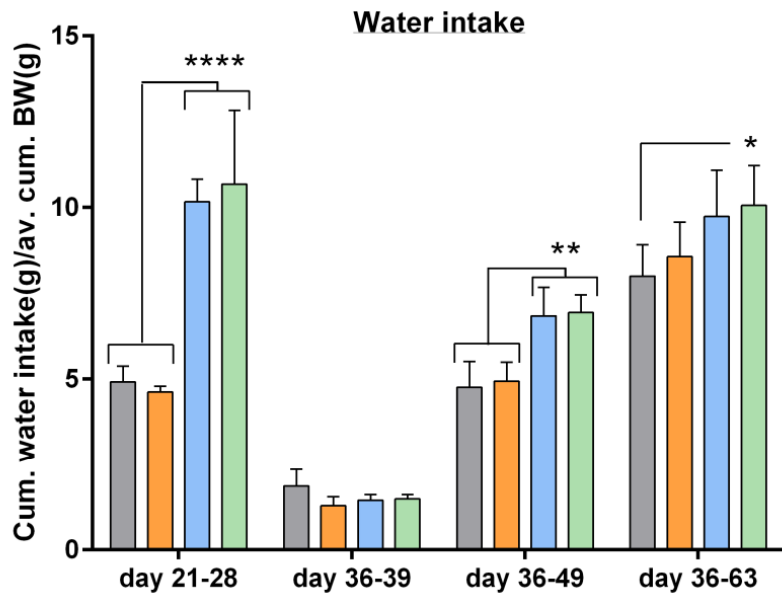

C

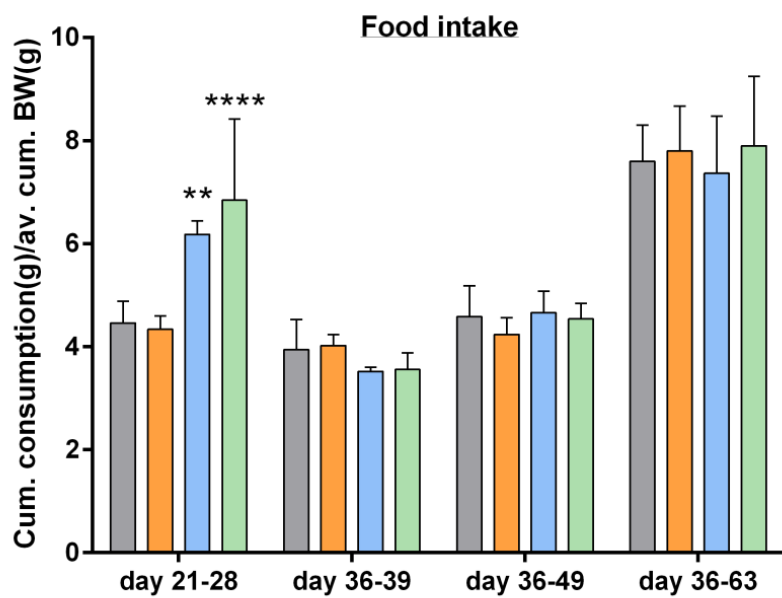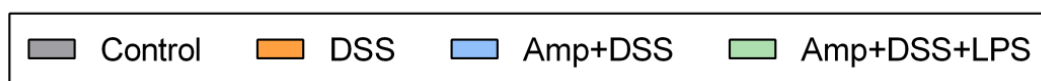

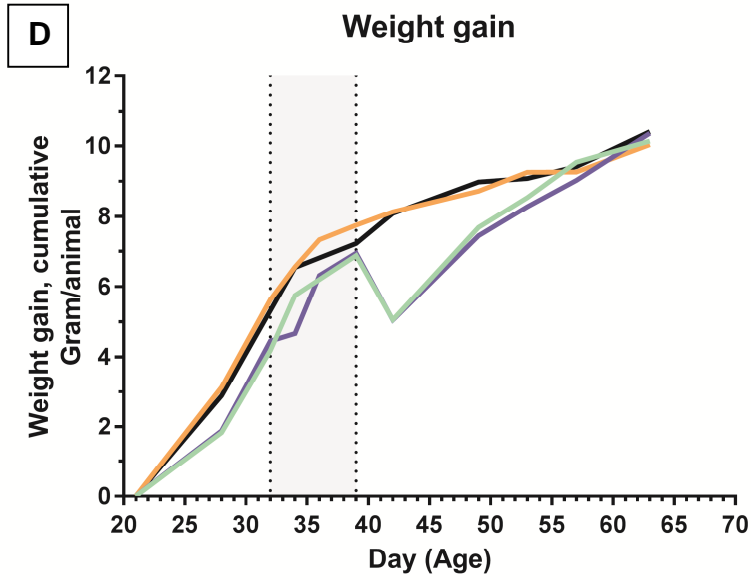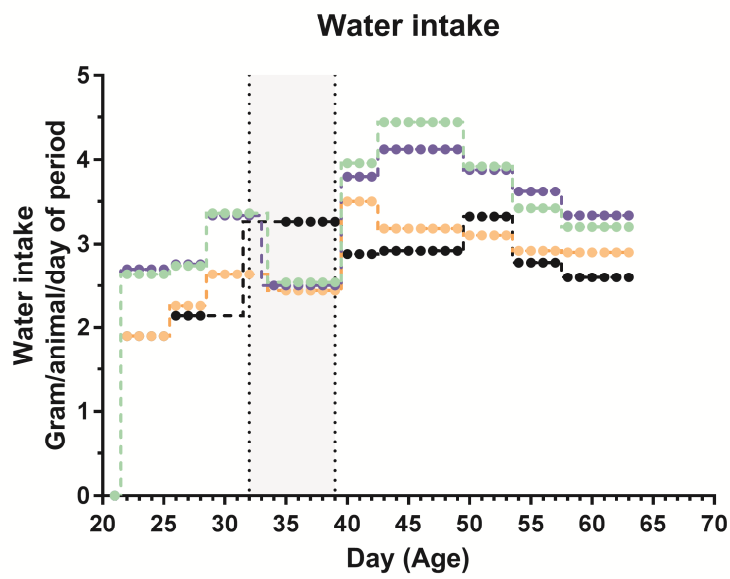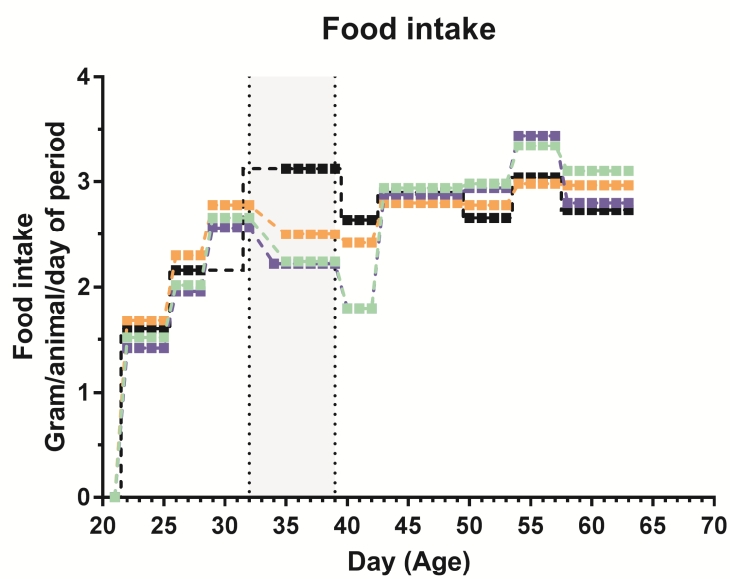

— DSS+Ampicillin+LPS  
— DSS+Ampicillin  
— DSS  
— Control

Treatment  
with DSS  
or DSS+LPS

Supplement: S1 File — Data of mice during, and 1 and 25 days post treatment with 1.5% dextran sulfate sodium (DSS), 1g/L ampicillin and/or diet containing 40.8 mg/kg lipopolysaccharides (LPS). Figure A. Average DSS load (mg/g BW, calculated from cage level water intake per mouse in cage) in the different treatment groups during treatment, in week 5 (day 32–39). Ampicillin in the water increased the water intake, and thereby DSS load in groups co-treated with ampicillin. Figures B+C. Food and water intake divided in periods. Animals were treated from day 32–39. Day 21–28: First week of acclimatization (ampicillin-treated groups were acclimatized to ampicillin water from arrival). Day 36–49: From day 5 of treatment to 10 days post-treatment. Day 36–63: From day 5 of treatment to day 25 post-treatment. Significantly increased water intake in groups receiving ampicillin (B), and food intake was significantly increased in the first week of acclimatization (C). Figure D. Overview curves of overall cumulative weight gain, water and food intake. Food and water intake was measured on cage level and divided with the number of animals per cage. The mice were weighed individually, but for these graphs the average weight of the animals in one cage was used to calculate the average intake per mouse. Control and DSS show similar patterns characterized by decrease in food intake, rise in water intake after treatment period (shaded area). DSS+Amp and DSS+Amp+LPS had lower water and food intake during treatment compared to control and DSS animals, with changes continuing in the period following treatment cessation, where the mice consumed less food and lost weight, and elicited increased water intake. (PDF) [file pone.0176662.s003.pdf]
