# Supplementary material for: Immunological effects of reduced mucosal integrity in the early life of BALB/c mice
Source: PLoS One. 2017 May 1;12(5):e0176662. doi: 10.1371/journal.pone.0176662 (PMC5411035; doi:10.1371/journal.pone.0176662)
Supplement: S1 Table — (DOCX) [file pone.0176662.s004.docx]

**S1 Table.** **P-values for mRNA transcripts with <2 or >2 fold change.**

| **Colon mRNA** | | | | | |
| --- | --- | --- | --- | --- | --- |
| **Time** | Treatment | Primer^a^ | p | Result | Ajdusted p |
| Day 25 pt | DSS-Amp | FOXP3_M18 | 0,0001 | significant | 0,0051 |
| Day 25 pt | DSS-Amp | IL1A_M38 | 0,0019 | significant | 0,0175 |
| Day 3 | DSS-Amp | IL-10_M28 | 0,0001 | significant | 0,0041 |
| Day 3 | DSS-Amp | MUC2_M55 | 0,0009 | significant | 0,0159 |
| Day 3 | DSS-Amp-LPS | IL-10_M28 | 0,0018 | significant | 0,0175 |
| Day 3 | DSS-Amp | MUC2_M57 | 0,0040 | significant | 0,0319 |
| Day 3 | DSS-Amp | IL-2_M40 | 0,0270 | significant | 0,0884 |
| Day 3 | DSS-Amp-LPS | MUC2_M57 | 0,0307 | significant | 0,0906 |
| Day 1 pt | DSS+Amp | IL1A_M38 | 0,0002 | significant | 0,0051 |
| Day 1 pt | DSS+Amp | IL-2_M40 | 0,0012 | significant | 0,0172 |
| Day 1 pt | DSS+Amp+LPS | IL-2_M40 | 0,0019 | significant | 0,0175 |
| Day 1 pt | DSS+Amp+LPS | IL1A_M38 | 0,0065 | significant | 0,0395 |
| Day 1 pt | DSS+Amp | MUC1_M53 | 0,0066 | significant | 0,0395 |
| Day 1 pt | DSS | HAPTOGLOBIN_M23 | 0,0084 | significant | 0,0443 |
| Day 1 pt | DSS+Amp+LPS | IL1BETA_M39 | 0,0086 | significant | 0,0443 |
| Day 1 pt | DSS+Amp+LPS | HAPTOGLOBIN_M23 | 0,0110 | tendency | 0,0527 |
| Day 1 pt | DSS | IL1A_M38 | 0,0132 | tendency | 0,0596 |
| Day 1 pt | DSS+Amp | FOXP3_M18 | 0,0153 | tendency | 0,0646 |
| Day 1 pt | DSS+Amp+LPS | IFNY_M27 | 0,0174 | tendency | 0,0695 |
| Day 1 pt | DSS+Amp+LPS | IL-6_M47 | 0,0184 | tendency | 0,0696 |
| Day 1 pt | DSS | IL1BETA_M39 | 0,0225 | tendency | 0,0811 |
| Day 1 pt | DSS | IL-2_M40 | 0,0261 | tendency | 0,0884 |
| Day 1 pt | DSS+Amp | MUC1_M54 | 0,0283 | tendency | 0,0885 |
| Day 1 pt | DSS+Amp+LPS | IFNY_M25 | 0,0318 | tendency | 0,0906 |
| Day 1 pt | DSS+Amp+LPS | CXCL9_M13 | 0,0327 | tendency | 0,0906 |
| Day 1 pt | DSS+Amp | IL1BETA_M39 | 0,0365 | tendency | 0,0972 |
| Day 3 | DSS | DEFENSIN-A_M94 | 0,0440 | ns | 0,1132 |
| Dag 25 pt | DSS-Amp-LPS | IL1A_M38 | 0,0491 | ns | 0,1219 |
| Day 3 | DSS-Amp-LPS | HAPTOGLOBIN_M23 | 0,0602 | ns | 0,1403 |
| Day 3 | DSS-Amp-LPS | MUC2_M55 | 0,0604 | ns | 0,1403 |
| Day 3 | DSS-Amp-LPS | IL-2_M40 | 0,1035 | ns | 0,2320 |
| Day 3 | DSS-Amp | RELMB_M65 | 0,1112 | ns | 0,2320 |
| Day 25 pt | DSS-Amp | MUC1_M53 | 0,1112 | ns | 0,2320 |
| Day 3 | DSS-Amp | RELMB_M121 | 0,1136 | ns | 0,2320 |
| Day 3 | DSS | MUC2_M57 | 0,1198 | ns | 0,2320 |
| Day 3 | DSS-Amp-LPS | IL-6_M47 | 0,1215 | ns | 0,2320 |
| Day 3 | DSS | MUC2_M55 | 0,1225 | ns | 0,2320 |
| Day 3 | DSS | IL_23_M41 | 0,1272 | ns | 0,2348 |
| Day 25 pt | DSS-Amp-LPS | RELMB_M121 | 0,1525 | ns | 0,2744 |
| Day 25 pt | DSS | RELMB_M65 | 0,1658 | ns | 0,2851 |
| Day 3 | DSS | IL-2_M40 | 0,1671 | ns | 0,2851 |
| Day 25 pt | DSS | RELMB_M121 | 0,1703 | ns | 0,2851 |
| Day 3 | DSS | IL-10_M28 | 0,1785 | ns | 0,2912 |
| Day 25 pt | DSS-Amp-LPS | RELMB_M65 | 0,1821 | ns | 0,2912 |
| Day 25 pt | DSS-Amp | RELMB_M121 | 0,1861 | ns | 0,2912 |
| Day 25 pt | DSS | FOXP3_M18 | 0,2003 | ns | 0,3061 |
| Day 3 | DSS | HAPTOGLOBIN_M23 | 0,2041 | ns | 0,3061 |
| Day 25 pt | DSS-Amp | RELMB_M65 | 0,2184 | ns | 0,3209 |
| Day 25 pt | DSS-Amp-LPS | MUC1_M53 | 0,2390 | ns | 0,3441 |
| Day 3 | DSS-Amp | TIMP_M75 | 0,2537 | ns | 0,3581 |
| Day 3 | DSS-Amp | IL-6_M47 | 0,2810 | ns | 0,3891 |
| Day 3 | DSS-Amp | HAPTOGLOBIN_M23 | 0,2864 | ns | 0,3891 |
| Day 3 | DSS | IL-6_M47 | 0,2964 | ns | 0,3932 |
| Day 3 | DSS-Amp | IL_23_M41 | 0,3003 | ns | 0,3932 |
| Day 3 | DSS | RELMB_M65 | 0,3198 | ns | 0,4112 |
| Day 3 | DSS | RELMB_M121 | 0,3365 | ns | 0,4250 |
| Day 25 pt | DSS-Amp-LPS | ALPI_M3 | 0,3530 | ns | 0,4357 |
| Day 3 | DSS-Amp-LPS | TIMP_M75 | 0,3570 | ns | 0,4357 |
| Day 3 | DSS-Amp-LPS | RELMB_M65 | 0,3752 | ns | 0,4502 |
| Day 3 | DSS-Amp-LPS | RELMB_M121 | 0,3877 | ns | 0,4576 |
| Day 25 pt | DSS | ALPI_M3 | 0,4352 | ns | 0,5054 |
| Day 25 pt | DSS-Amp | ALPI_M3 | 0,4613 | ns | 0,5272 |
| Day 3 | DSS-Amp-LPS | DEFENSIN-A_M94 | 0,4915 | ns | 0,5506 |
| Day 25 pt | DSS | IL1A_M38 | 0,4970 | ns | 0,5506 |
| Day 3 | DSS-Amp | DEFENSIN-A_M94 | 0,5457 | ns | 0,5954 |
| Day 25 pt | DSS | DEFENSIN-A_M94 | 0,6797 | ns | 0,7304 |
| Day 25 pt | DSS | MUC1_M53 | 0,7176 | ns | 0,7598 |
| Day 3 | DSS-Amp-LPS | IL_23_M41 | 0,7325 | ns | 0,7644 |
| Day 25 pt | DSS-Amp-LPS | DEFENSIN-A_M94 | 0,7834 | ns | 0,8058 |
| Day 25 pt | DSS-Amp | DEFENSIN-A_M94 | 0,8106 | ns | 0,8220 |
| Day 3 | DSS | TIMP_M75 | 0,8413 | ns | 0,8413 |
| **Ileum mRNA** | | | | | |
| Time | Treatment | Gene | p | Result | Adjusted p |
| Day 1 pt | DSS+Amp | IFNY_M27 | 0,0001 | significant | 0,0027 |
| Day 1 pt | DSS | RELMB_M65 | 0,0001 | significant | 0,0027 |
| Day 1 pt | DSS | SAA_M71 | 0,0001 | significant | 0,0029 |
| Day 1 pt | DSS+Amp | CXCL9_M13 | 0,0002 | significant | 0,0039 |
| Day 1 pt | DSS+Amp | IL-10_M28 | 0,0005 | significant | 0,0070 |
| Day 1 pt | DSS+Amp+LPS | IFNY_M27 | 0,0013 | significant | 0,0144 |
| Day 1 pt | DSS+Amp | IFNY_M25 | 0,0014 | significant | 0,0144 |
| Day 1 pt | DSS+Amp | CXCL10_M10 | 0,0018 | significant | 0,0158 |
| Day 1 pt | DSS-Amp | RELMB_M65 | 0,0021 | significant | 0,0158 |
| Day 1 pt | DSS+Amp+LPS | IFNY_M25 | 0,0022 | significant | 0,0158 |
| Day 1 pt | DSS+Amp+LPS | CXCL9_M13 | 0,0039 | significant | 0,0257 |
| Day 1 pt | DSS+Amp+LPS | IL-6_M47 | 0,0057 | significant | 0,0343 |
| Day 1 pt | DSS+Amp+LPS | IL-10_M28 | 0,0098 | tendency | 0,0508 |
| Day 3 | DSS-Amp | SAA_M71 | 0,0099 | tendency | 0,0508 |
| Day 25 pt | DSS+Amp | IL-18_M36 | 0,0124 | tendency | 0,0594 |
| Day 3 | DSS | SAA_M71 | 0,0138 | tendency | 0,0623 |
| Day 1 pt | DSS+Amp+LPS | CXCL10_M10 | 0,0205 | tendency | 0,0868 |
| Day 3 | DSS+Amp+LPS | IL-10_M28 | 0,0244 | tendency | 0,0968 |
| Day 3 | DSS+Amp+LPS | RELMB_M65 | 0,0255 | tendency | 0,0968 |
| Day 25 pt | DSS+Amp+LPS | IFNY_M27 | 0,0278 | ns | 0,1001 |
| Day 1 pt | DSS+Amp+LPS | SAA_M71 | 0,0336 | ns | 0,1152 |
| Day 25 pt | DSS+Amp+LPS | IL-18_M36 | 0,0447 | ns | 0,1464 |
| Day 3 | DSS-Amp | IL-10_M28 | 0,0529 | ns | 0,1639 |
| Day 1 pt | DSS | IL-12_M33 | 0,0546 | ns | 0,1639 |
| Day 3 | DSS | TNFSF15_M80 | 0,0569 | ns | 0,1640 |
| Day 25 pt | DSS+Amp+LPS | IFNY_M25 | 0,0646 | ns | 0,1756 |
| Day 3 | DSS | IL-10_M28 | 0,0665 | ns | 0,1756 |
| Day 1 pt | DSS | IL-10_M28 | 0,0696 | ns | 0,1756 |
| Day 3 | DSS+Amp+LPS | SAA_M71 | 0,0707 | ns | 0,1756 |
| Day 1 pt | DSS+Amp+LPS | IL-12_M31 | 0,0841 | ns | 0,2019 |
| Day 1 pt | DSS+Amp | IL-12_M31 | 0,0905 | ns | 0,2101 |
| Day 3 | DSS+Amp+LPS | IFNY_M25 | 0,1003 | ns | 0,2256 |
| Day 1 pt | DSS | IL-12_M31 | 0,1034 | ns | 0,2256 |
| Day 1 pt | DSS+Amp | SAA_M71 | 0,1157 | ns | 0,2379 |
| Day 1 pt | DSS | IFNY_M25 | 0,1188 | ns | 0,2379 |
| Day 1 pt | DSS | TNFSF15_M80 | 0,1205 | ns | 0,2379 |
| Day 1 pt | DSS | IFNY_M27 | 0,1223 | ns | 0,2379 |
| Day 3 | DSS | RELMB_M65 | 0,1348 | ns | 0,2554 |
| Day 1 pt | DSS+Amp | IL-6_M47 | 0,1435 | ns | 0,2650 |
| Day 25 pt | DSS+Amp+LPS | IL-10_M28 | 0,1485 | ns | 0,2672 |
| Day 3 | DSS-Amp | IFNY_M25 | 0,1611 | ns | 0,2829 |
| Day 1 pt | DSS+Amp | IL-12_M33 | 0,1700 | ns | 0,2914 |
| Day 1 pt | DSS+Amp | TNFSF15_M80 | 0,1928 | ns | 0,3217 |
| Day 3 | DSS | IFNY_M25 | 0,1966 | ns | 0,3217 |
| Day 25 pt | DSS+Amp | IFNY_M27 | 0,2195 | ns | 0,3513 |
| Day 1 pt | DSS+Amp+LPS | IL-12_M33 | 0,2620 | ns | 0,4032 |
| Day 25 pt | DSS+Amp | IFNY_M25 | 0,2632 | ns | 0,4032 |
| Day 3 | DSS | HAPTOGLOBIN_M23 | 0,2729 | ns | 0,4093 |
| Day 3 | DSS+Amp+LPS | HAPTOGLOBIN_M23 | 0,2987 | ns | 0,4389 |
| Day 1 pt | DSS | CXCL9_M13 | 0,3082 | ns | 0,4438 |
| Day 1 pt | DSS | CXCL10_M10 | 0,3406 | ns | 0,4808 |
| Day 1 pt | DSS+Amp+LPS | RELMB_M65 | 0,3605 | ns | 0,4991 |
| Day 3 | DSS-Amp | TNFSF15_M80 | 0,3995 | ns | 0,5427 |
| Day 25 pt | DSS | IL-18_M36 | 0,4177 | ns | 0,5569 |
| Day 25 pt | DSS+Amp+LPS | TNFSF15_M80 | 0,4469 | ns | 0,5850 |
| Day 25 pt | DSS | RELMB_M65 | 0,5030 | ns | 0,6391 |
| Day 1 pt | DSS+Amp | RELMB_M65 | 0,5059 | ns | 0,6391 |
| Day 25 pt | DSS | IFNY_M25 | 0,5253 | ns | 0,6521 |
| Day 25 pt | DSS | TNFSF15_M80 | 0,5407 | ns | 0,6599 |
| Day 3 | DSS+Amp+LPS | TNFSF15_M80 | 0,5904 | ns | 0,7085 |
| Day 1 pt | DSS | HAPTOGLOBIN_M23 | 0,6158 | ns | 0,7269 |
| Day 1 pt | DSS+Amp+LPS | TNFSF15_M80 | 0,6282 | ns | 0,7295 |
| Day 1 pt | DSS+Amp+LPS | HAPTOGLOBIN_M23 | 0,6675 | ns | 0,7628 |
| Day 25 pt | DSS+Amp+LPS | RELMB_M65 | 0,6906 | ns | 0,7752 |
| Day 25 pt | DSS+Amp | IL-10_M28 | 0,6999 | ns | 0,7752 |
| Day 3 | DSS-Amp | HAPTOGLOBIN_M23 | 0,7267 | ns | 0,7914 |
| Day 25 pt | DSS+Amp | TNFSF15_M80 | 0,7364 | ns | 0,7914 |
| Day 25 pt | DSS | IFNY_M27 | 0,7588 | ns | 0,8035 |
| Day 25 pt | DSS | IL-10_M28 | 0,7770 | ns | 0,8063 |
| Day 1 pt | DSS | IL-6_M47 | 0,7839 | ns | 0,8063 |
| Day 1 pt | DSS+Amp | HAPTOGLOBIN_M23 | 0,9307 | ns | 0,9438 |
| Day 25 pt | DSS+Amp | RELMB_M65 | 0,9577 | ns | 0,9577 |

Evaluated in mice on day 3, and on day 1 and 25 days post treatment with 1.5% dextran sulfate sodium (DSS), 1g/L ampicillin and/or diet containing 40.8 mg/kg lipopolysaccharides (LPS) compared to control mice.

T-test on log2 values, against control group, FDR-corrected (Benjamini-Hochberg). ^a^M-numbers; primer specific.
