# Supplementary material for: Immunological effects of reduced mucosal integrity in the early life of BALB/c mice
Source: PLoS One. 2017 May 1;12(5):e0176662. doi: 10.1371/journal.pone.0176662 (PMC5411035; doi:10.1371/journal.pone.0176662)
Supplement: S2 Table — (DOCX) [file pone.0176662.s005.docx]

**S2 Table. Analysis of variance of gut microbiota.**

| **Between time points** |  |  |  |  | Abundance |  | ANOVA |  |  |
| --- | --- | --- | --- | --- | --- | --- | --- | --- | --- |
| *Phylum* | *Class* | *Order* | *Family* | *Genus* | ***Day 3 (%)*** | ***Day 1 pt(%)*** | *p* | *Bonfer* | *FDR* |
| **Control** |  |  |  |  |  |  |  |  |  |
| Bacteroidetes | Bacteroidia | Bacteroidales | Rikenellaceae |  | 18,624 | 41,603 | 0,016 | 0,535 | 0,535 |
| Firmicutes | Clostridia | Clostridiales |  |  | 39,148 | 20,015 | 0,029 | 0,997 | 0,498 |
| Firmicutes | Clostridia | Clostridiales | Lachnospiraceae | Coprococcus | 0,285 | 0,079 | 0,030 | 1,019 | 0,340 |
| Proteobacteria | Gammaproteobacteria | Enterobacteriales | Enterobacteriaceae | Other | 0,037 | 0,000 | 0,036 | 1,232 | 0,308 |
|  |  |  |  |  | ***Day 1 pt (%)*** | ***Day 25 pt(%)*** | *p* | *Bonfer* | *FDR* |
| Bacteroidetes | Bacteroidia | Bacteroidales | Rikenellaceae |  | 41,611 | 18,053 | 0,030 | 1,066 | 1,066 |
| Bacteroidetes | Bacteroidia | Bacteroidales | S24-7 |  | 12,115 | 33,944 | 0,035 | 1,232 | 0,616 |
| **DSS** |  |  |  |  |  |  |  |  |  |
|  |  |  |  |  | ***Day 3 (%)*** | ***Day 1 pt(%)*** | *p* | *Bonfer* | *FDR* |
| Proteobacteria | Gammaproteobacteria | Enterobacteriales | Enterobacteriaceae | Other | 0,033 | 0,000 | 0,010 | 0,212 | 0,212 |
| Firmicutes | Clostridia | Clostridiales | Ruminococcaceae | Oscillospira | 2,852 | 0,242 | 0,033 | 0,718 | 0,359 |
|  |  |  |  |  | ***Day 1 pt (%)*** | ***Day 25 pt(%)*** | *p* | *Bonfer* | *FDR* |
| ***Firmicutes*** | ***Clostridia*** | ***Clostridiales*** | ***Ruminococcaceae*** | ***Oscillospira*** | *0,240* | *2,696* | *0,001* | *0,043* | *0,043* |
| ***Firmicutes*** | ***Clostridia*** | ***Clostridiales*** | ***Lachnospiraceae*** |  | *0,110* | *2,087* | *0,001* | *0,045* | *0,022* |
| ***Bacteroidetes*** | ***Bacteroidia*** | ***Bacteroidales*** | ***Rikenellaceae*** |  | *1,121* | *18,200* | *0,003* | *0,089* | *0,030* |
| Firmicutes | Clostridia | Clostridiales | Ruminococcaceae |  | 0,240 | 1,566 | 0,010 | 0,289 | 0,072 |
| Bacteroidetes | Bacteroidia | Bacteroidales | Prevotellaceae | Prevotella | 0,000 | 0,762 | 0,011 | 0,333 | 0,067 |
| Firmicutes | Clostridia | Clostridiales | Ruminococcaceae | Other | 0,000 | 0,019 | 0,013 | 0,380 | 0,063 |
| Firmicutes | Clostridia | Clostridiales | Clostridiaceae | CandidatusArthromitus | 0,002 | 0,066 | 0,019 | 0,562 | 0,080 |
| Firmicutes | Clostridia | Clostridiales | Lachnospiraceae | Other | 0,000 | 0,052 | 0,020 | 0,611 | 0,076 |
| Cyanobacteria | 4C0d-2 | YS2 |  |  | 0,005 | 0,366 | 0,024 | 0,724 | 0,080 |
| Bacteroidetes | Bacteroidia | Bacteroidales | Bacteroidaceae | Bacteroides | 68,905 | 40,047 | 0,030 | 0,912 | 0,091 |
| Firmicutes | Clostridia | Clostridiales | Ruminococcaceae | Ruminococcus | 0,013 | 0,159 | 0,041 | 1,236 | 0,112 |
| **Between groups** |  |  |  |  |  |  |  |  |  |
| *Phylum* | *Class* | *Order* | *Family* | *Genus* | ***Control (%)*** | ***DSS (%)*** | *p* | *Bonfer* | *FDR* |
| **Day 3** |  |  |  |  |  |  |  |  |  |
| *Bacteroidetes* | *Bacteroidia* | *Bacteroidales* | *Rikenellaceae* |  | *18,620* | *2,583* | *0,000* | *0,004* | *0,004* |
| *Bacteroidetes* | *Bacteroidia* | *Bacteroidales* | *Bacteroidaceae* | *Bacteroides* | *9,207* | *58,967* | *0,001* | *0,021* | *0,010* |
| *Firmicutes* | *Clostridia* | *Clostridiales* | *Clostridiaceae* | *Candidatus Arthromitus* | *0,244* | *0,004* | *0,002* | *0,054* | *0,018* |
| *Firmicutes* | *Clostridia* | *Clostridiales* |  |  | *39,155* | *5,210* | *0,002* | *0,062* | *0,016* |
| **Day 1 pt** |  |  |  |  |  |  |  |  |  |
| *Bacteroidetes* | *Bacteroidia* | *Bacteroidales* | *Bacteroidaceae* | *Bacteroides* | *6,867* | *68,896* | *0,000* | *0,000* | *0,000* |
| *Bacteroidetes* | *Bacteroidia* | *Bacteroidales* | *Rikenellaceae* |  | *41,606* | *1,121* | *0,000* | *0,006* | *0,003* |
| *Firmicutes* | *Clostridia* | *Clostridiales* | *Lachnospiraceae* | *Other* | *0,078* | *0,000* | *0,004* | *0,110* | *0,037* |
| *Firmicutes* | *Bacilli* | *Lactobacillales* | *Lactobacillaceae* | *Lactobacillus* | *0,565* | *0,106* | *0,005* | *0,144* | *0,036* |
| *Firmicutes* | *Clostridia* | *Clostridiales* | *[Mogibacteriaceae]* |  | *0,038* | *0,002* | *0,006* | *0,191* | *0,038* |
| **Day 25 pt** |  |  |  |  |  |  |  |  |  |
| Bacteroidetes | Bacteroidia | Bacteroidales | S24-7 |  | 33,940 | 6,788 | 0,005 | 0,163 | 0,163 |
| Bacteroidetes | Bacteroidia | Bacteroidales | Bacteroidaceae | Bacteroides | 6,049 | 40,049 | 0,014 | 0,447 | 0,223 |
|  |  |  |  |  | ***DSS (%)*** | ***DSS+Amp(+LPS) (%)*** | *p* | *Bonfer* | *FDR* |
| *Bacteroidetes* | *Bacteroidia* | *Bacteroidales* | *Rikenellaceae* |  | *18,197* | *0,501* | *0,001* | *0,026* | *0,026* |
| *Bacteroidetes* | *Bacteroidia* | *Bacteroidales* | *[Odoribacteraceae]* | *Odoribacter* | *0,320* | *0,000* | *0,002* | *0,058* | *0,029* |
| *Bacteroidetes* | *Bacteroidia* | *Bacteroidales* |  |  | *1,135* | *0,001* | *0,005* | *0,146* | *0,049* |
| *Firmicutes* | *Bacilli* | *Lactobacillales* | *Lactobacillaceae* | *Lactobacillus* | *0,239* | *0,002* | *0,005* | *0,154* | *0,039* |
| *Bacteroidetes* | *Bacteroidia* | *Bacteroidales* | *Prevotellaceae* | *Prevotella* | *0,761* | *0,000* | *0,005* | *0,174* | *0,035* |
|  |  |  |  |  | ***Control (%)*** | ***DSS+Amp(+LPS) (%)*** | *p* | *Bonfer* | *FDR* |
| *Cyanobacteria* | *4C0d-2* | *YS2* |  |  | *0,356* | *0,000* | *0,000* | *0,002* | *0,001* |
| *Bacteroidetes* | *Bacteroidia* | *Bacteroidales* | *Prevotellaceae* | *Prevotella* | *0,306* | *0,001* | *0,000* | *0,004* | *0,001* |
| *Bacteroidetes* | *Bacteroidia* | *Bacteroidales* | *Rikenellaceae* |  | *18,802* | *1,236* | *0,000* | *0,012* | *0,003* |
| *Bacteroidetes* | *Bacteroidia* | *Bacteroidales* | *Porphyromonadaceae* | *Parabacteroides* | *0,174* | *0,004* | *0,000* | *0,012* | *0,002* |
| *Bacteroidetes* | *Bacteroidia* | *Bacteroidales* | *[Odoribacteraceae]* | *Odoribacter* | *0,410* | *0,000* | *0,000* | *0,014* | *0,002* |
| *Bacteroidetes* | *Bacteroidia* | *Bacteroidales* |  |  | *2,160* | *0,002* | *0,009* | *0,264* | *0,038* |

Analysis of variance of gut microbiota in mice on day 3, and on day 1 and 25 days post treatment with 1.5% dextran sulfate sodium (DSS), 1g/L ampicillin and/or diet containing 40.8 mg/kg lipopolysaccharides (LPS) (S3 Fig).
