# Supplementary material for: Immunological effects of reduced mucosal integrity in the early life of BALB/c mice
Source: PLoS One. 2017 May 1;12(5):e0176662. doi: 10.1371/journal.pone.0176662 (PMC5411035; doi:10.1371/journal.pone.0176662)
Supplement: S3 Table — (DOCX) [file pone.0176662.s006.docx]

**S3 Table. RNA Integrity.**

|  |  |  | Ileum | | Colon | |
| --- | --- | --- | --- | --- | --- | --- |
| Day | ID | Group | RIN | Sample | RIN | Sample |
| 25 pt | 2 | _DSS+Amp_ | 9.6 | 2 | 7.3 | 3 |
|  | 42 | _DSS+Amp+LPS_ | 9.5 | 5 | 7.8 | 6 |
|  | 119 | _DSS+Amp_ | 8.7 | 8 | 7.1 | 9 |
|  | 41 | _DSS+Amp_ | 8.7 | 11 | 9.1 | 12 |
|  | 100 | _DSS+Amp_ | 8.7 | 14 | 9.8 | 15 |
|  | 106 | _DSS+Amp+LPS_ | 8.9 | 17 | 9.8 | 18 |
|  | 99 | _DSS+Amp+LPS_ | 8.5 | 20 | 8.8 | 21 |
|  | 88 | _Control_ | 8.2 | 23 | 8.0 | 24 |
|  | 84 | _DSS+Amp+LPS_ | 7.8 | 26 | 8.4 | 27 |
|  | 15 | _Control_ | 9.9 | 29 | 9.5 | 30 |
|  | 112 | _Control_ | 9.2 | 32 | 8.6 | 33 |
|  | 33 | _DSS+Amp+LPS_ | 8.9 | 38 | 7.9 | 39 |
|  | 80 | _DSS+Amp+LPS_ | 8.3 | 41 | 8.3 | 42 |
|  | 52 | _Control_ | 9.8 | 44 | 8.4 | 45 |
|  | 65 | _Control_ | 9.7 | 183 | 8.9 | 164 |
|  | 9 | _DSS_ | 9.7 | 47 | 9.2 | 48 |
|  | 102 | _DSS+Amp_ | 9.0 | 50 | 8.9 | 51 |
|  | 13 | _DSS_ | 9.1 | 53 | 6.6 | 54 |
|  | 46 | _Control_ | 8.9 | 56 | 8.9 | 57 |
|  | 96 | _DSS_ | 9.9 | 59 | 7.5 | 60 |
|  | 54 | _DSS_ | 9.8 | 62 | 9.2 | 63 |
|  | 44 | _DSS_ | 8.9 | 65 | 8.6 | 66 |
|  | 57 | _DSS+Amp_ | 9.3 | 68 | 8.8 | 69 |
|  | 93 | _DSS_ | 9.8 | 35 | 9.2 | 36 |
| 3 | 45 | _DSS+Amp+LPS_ | 9.1 | 122 | 9.2 | 123 |
|  | 70 | _DSS+Amp+LPS_ | 9.3 | 119 | 8.8 | 120 |
|  | 77 | _DSS+Amp+LPS_ | 9.3 | 116 | 9.2 | 117 |
|  | 85 | _Control_ | 9.6 | 113 | 8.5 | 114 |
|  | 66 | _DSS+Amp+LPS_ | 10.0 | 161 | 9.4 | 162 |
|  | 51 | _Control_ | 9.3 | 149 | 9.4 | 350 |
|  | 18 | _Control_ | 9.4 | 152 | 9.6 | 153 |
|  | 115 | _DSS_ | 8.9 | 137 | 8.7 | 138 |
|  | 23 | _DSS_ | 9.6 | 134 | 9.2 | 135 |
|  | 72 | _DSS+Amp_ | 9.1 | 125 | 8.8 | 126 |
|  | 71 | _DSS_ | 9.6 | 131 | 8.9 | 132 |
|  | 24 | _DSS+Amp_ | 9.2 | 128 | 9.6 | 129 |
|  | 89 | _DSS+Amp_ | 9.3 | 178 | 9.6 | 390 |
|  | 120 | _DSS_ | 9.6 | 140 | 8.5 | 141 |
|  | 43 | _DSS_ | 9.2 | 143 | 8.7 | 144 |
|  | 68 | _Control_ | 9.3 | 146 | 8.5 | 147 |
|  | 17 | _DSS+Amp_ | 9.9 | 158 | 9.5 | 159 |
|  | 48 | _DSS+Amp_ | 9.1 | 182 | 9.3 | 192 |
|  | 14 | _Control_ | 9.8 | 155 | 9.3 | 156 |
|  | 98 | _DSS+Amp+LPS_ | 9.4 | 180 | 9.2 | 191 |
| 1 pt | 101 | _DSS_ | 9.3 | 166 | 9.6 | 184 |
|  | 39 | _DSS+Amp_ | 9.3 | 168 | 9.2 | 185 |
|  | 8 | _DSS+Amp_ | 9.9 | 71 | 8.3 | 72 |
|  | 19 | _DSS+Amp+LPS_ | 9.9 | 176 | 9.3 | 189 |
|  | 1 | _DSS+Amp+LPS_ | 9.5 | 174 | 9.6 | 188 |
|  | 117 | _DSS+Amp+LPS_ | 9.1 | 107 | 9.2 | 108 |
|  | 81 | _DSS+Amp_ | 9.3 | 172 | 9.2 | 187 |
|  | 22 | _Control_ | 9.8 | 74 | 9.0 | 75 |
|  | 32 | _Control_ | 9.9 | 89 | 9.3 | 90 |
|  | 47 | _DSS_ | 9.7 | 77 | 8.9 | 78 |
|  | 104 | _Control_ | 9.8 | 80 | 9.3 | 81 |
|  | 34 | _DSS+Amp+LPS_ | 9.6 | 92 | 9.4 | 93 |
|  | 90 | _DSS+Amp_ | 9.4 | 95 | 9.6 | 96 |
|  | 35 | _Control_ | 9.9 | 83 | 9.7 | 84 |
|  | 83 | _DSS+Amp+LPS_ | 9.5 | 86 | 7.2 | 87 |
|  | 38 | _DSS_ | 9.4 | 98 | 9.1 | 99 |
|  | 105 | _DSS_ | 9.7 | 101 | 6.9 | 102 |
|  | 59 | _DSS+Amp_ | 9.5 | 170 | 9.2 | 186 |
|  | 92 | _DSS_ | 9.4 | 104 | 9.4 | 105 |
|  | 56 | _Control_ | 9.6 | 110 | 9.5 | 111 |

RNA extracted from mice on day 3, and on day 1 and 25 days post treatment with 1.5% dextran sulfate sodium (DSS), 1g/L ampicillin and/or diet containing 40.8 mg/kg lipopolysaccharides (LPS).
