# Supplementary material for: Immunological effects of reduced mucosal integrity in the early life of BALB/c mice
Source: PLoS One. 2017 May 1;12(5):e0176662. doi: 10.1371/journal.pone.0176662 (PMC5411035; doi:10.1371/journal.pone.0176662)
Supplement: S4 Table — (DOCX) [file pone.0176662.s007.docx]

**S4 Table**. **Primers**.

| *Gene symbol* | *Name (Abbreviation)* | *Forward sequence* | *Reverse sequence* | *Amplicon length* |
| --- | --- | --- | --- | --- |
| Foxp3 | Forkhead box P3 | CACCTGGAAGAATGCCATC | GTCCACACTGCTCCCTTCTC | 84 |
| Il10 | Interleukin-10 | AAAGGACCAGCTGGACAACA | TAAGGCTTGGCAACCCAAGTA | 79 |
| Il10rb | Interleukin receptor beta | CCATCATTGGACCTCCTGA | GTCCACGTCTCAGGCTCATT | 100 |
| Tgfb(1) | Transforming growth factor beta | GCTGCGCTTGCAGAGATTAA | GTAACGCCAGGAATTGTTGCTA | 82 |
| Tgfb(2) |  | TGAGTGGCTGTCTTTTGACG | AGCCCTGTATTCCGTCTCCT | 71 |
| Stat5 | signal transducer and activator of transcription 5 | GGTCCCTGAGTTCGTCAATG | GGTTGGGTGGGTACATGTTG | 116 |
| Tnfa | Tumor necrosis factor alpha | CAAATGGCCTCCCTCTCATCA | TGGGCTACAGGCTTGTCAC | 88 |
| Nfrkb | Nuclear factor kappa b | GGCAGGTATTTGACATACTAAATGG | TGCAGAGTTGTAGCCTCGTG | 117 |
| Il12 (1) | Interleukin-12 | AAACCAGCACATTGAAGACC | GGAAGAAGTCTCTCTAGTAGCC | 80 |
| Il12 (2) |  | AGCACTTCAGAATCACAACCATC | GAGGTTTCTGGCGCAGAGT | 116 |
| Il6 | Interleukin-6 | CTGGAGTACCATAGCTACCTGGAG | CTCTGAAGGACTCTGGCTTTG | 80 |
| Il1a | Interleukin-1 alpha | AGATGGCCAAAGTTCCTGAC | AGAGATGGTCAATGGCAGAAC | 87 |
| Ifng(1) | Interferon-gamma | GGCACAGTCATTGAAAGCCTA | GCCAGTTCCTCCAGATATCCA | 103 |
| Ifng (2) |  | ACTGGCAAAAGGATGGTGAC | GCTGATGGCCTGATTGTCTT | 98 |
| Il1b | Interleukin-1 beta | TGCAGCTGGAGAGTGTGG | TCAAACTCCACTTTGCTCTTGA | 100 |
| Tnf-sf15/  Tl1a | Tumor necrosis factor superfamily 15/ Tumor necrosis factor-like ligand 1A | GCAAGCCGAGAGCACAC | CCATCCCTAGGTCATGTTCCC | 99 |
| Il2 | Interleukin-2 | GTAAAACTAAAGGGCTCTGACAACAC | GGGCTTGTTGAGATGATGCT | 119 |
| Il18 (1) | Interleukin-18 | CAAAGAAAGCCGCCTCAAAC | GACGCAAGAGTCTTCTGACA | 82 |
| Il18 (2) |  | ACGTGTTCCAGGACACAACA | CGCTGCTGCCTTCACTGTA | 79 |
| Il23 | Interleukin-23 | CCTCCAGCCAGAGGATCAC | GAAGGATCTTGGAACGGAGA | 98 |
| Tlr4 (1) | Toll-like receptor 4 | GTTCTTCTCCTGCCTGACAC | GCTGAGTTTCTGATCCATGCA | 91 |
| Tlr4 (2) |  | CTTCAACCAAGAACATAGATCTGAGC | GTCTCCACAGCCACCAGATT | 247 |
| Tlr1 | Toll-like receptor 1 | CCACAAGCTCAAAACTCTCATGT | GCTCTTAGGTTTGCCCAAAAA | 93 |
| Md2/Ly96 | Lymphocyte antigen 96 | TTCCTAAGGGCCATTACAGATG | GCGGTGAATGATGGTGAAAT | 92 |
| Cd14 | CD14 antigen | CGTGTGCTTGGCTTGTTG | TGGCTTCGGATCTGAGAAGT | 111 |
| Muc1 (1) | Mucin 1 | TGGATTGTTTCTGCAGATTTTTAAC | AAAAGTACCCTCCCGGAAAA | 112 |
| Muc1 (2) |  | GAAGTCAAAGTGAATGAGATGCAG | ACGATAGCCAAAGCAACCAA | 116 |
| Muc2 (1) | Mucin 2 | TATGCCAGGCCAGGAGTTTA | GCAAGGCAGGTCTTTACACA | 82 |
| Muc2 (2) |  | GTGGGACTTTTGCCATGTACTC | CGGACACTGGTCTTCTCCTC | 85 |
| Defa | Defensin alpha | CTTCAAGAGGAATCGTTGAGAGAT | TCAGCGACAGCAGAGTGTGT | 123 |
| Timp | Tissue inhibitor of metalloproteinase | GGGGTGTGCACAGTGTTTC | GACCTGATCCGTCCACAAAC | 81 |
| Klf4 | Kruppel-like factor 4 | CGAGAAACCTTACCACTGTGACT | CCTGTGTGTTTGCGGTAGTG | 90 |
| Alpi | Alkaline phosphatase, intestinal | GTCACCGAAGCTCAGAGTGTT | GCAAATATGGCCACGTCCT | 98 |
| Retnlb (1) | Resistin like alpha | CTGTCCTGCTGGGATGGT | CCAGTCCATGACTGAGCACT | 109 |
| Retnlb (2) |  | GGTGGATCAAAGGATCAAGG | GTCTGCCAGAAGACGTGACA | 83 |
| Tff | Trefoil factor | CTGTCACATCGGAGCAGTGT | CAGGGCACATTTGGGATACT | 67 |
| Ocln | Occludin | GCTGCTGCTGATGAATATAATAGACT | TCCCACCATCCTCTTGATGT | 120 |
| Hp | Haptoglobin | TGAGGCAGTGTGTGGGAAG | TGGCGGGAGATCATCTTG | 114 |
| Saa | Serum amyloid A | GAGTCTGGGCTGCTGAGAAA | ATGGTGTCCTCGTGTCCTCT | 79 |
| Cxcl10 | Chemokine ligand 10 | AAGTGCTGCCGTCATTTTCT | CCTATGGCCCTCATTCTCAC | 129 |
| Ccl7 | Chemokine ligand 7 | GTGTCCCTGGGAAGCTGTTA | CTTTGGAGTTGGGGTTTTCA | 121 |
| Cxcl9 | Chemokine ligand 9 | AAGATCAGCCAAAAGAAAAAGC | GACGACTTTGGGGTGTTTTG | 88 |
| *Reference genes* | | | | |
| Tbp | TATA box binding protein | ACCAGAACAACAGCCTTCCA | AAAGATGGGAATTCCAGGAGTCA | 80 |
| Gusb | beta-glucuronidase | AAGAATACGTGGTCGGAGAGC | TCTCTGGCGAGTGAAGATCC | 107 |
| Hprt1 | hypoxanthine guanine phosphoribosyl transferase | CAGTACAGCCCCAAAATGGTTA | AGTCTGGCCTGTATCCAACA | 79 |
| Ppia | peptidylprolyl isomerase A | AAGACTGAATGGCTGGATGG | CATGGCTTCCACAATGTTCA | 75 |
| Rpl13a | ribosomal protein L13a | AGGTTACGGAAACAGGCAGAA | CAGGAGTCCGTTGGTCTTGA | 81 |
| B2m | beta-2 microglobulin | CTGGTGCTTGTCTCACTGAC | GGTGGGTGGCGTGAGTATA | 77 |
| Ywhae | tyrosine 3-monooxygenase/tryptophan 5-monooxygenase activation protein | CGGCAAATGGTTGAAACTG | TGGAATGAGGTGTTTGTCCA | 75 |
| Actb | beta-actin | CCCTAAGGCCAACCGTGAAA | CAGCCTGGATGGCTACGTAC | 83 |

RNA extracted from mice on day 3, and on day 1 and 25 days post treatment with 1.5% dextran sulfate sodium (DSS), 1g/L ampicillin and/or diet containing 40.8 mg/kg lipopolysaccharides (LPS).
